# Functional analysis of CYP4B1 enzymes from apes and humans uncovers evolutionary hot spots for adaptations of the catalytical function

**DOI:** 10.1371/journal.pgen.1011750

**Published:** 2025-06-27

**Authors:** Saskia Hüsken, Annika Röder, Johannes Ptok, Anne E. Meyer, Mats Georg, Yannick Schwarz, Christian Roos, Kerstin Mätz-Rensing, Michael C. Hutter, Doreen M. Floss, Allan E. Rettie, Marco Girhard, Helmut Hanenberg, Constanze Wiek

**Affiliations:** 1 Department of Otorhinolaryngology, Medical Faculty and University Hospital Düsseldorf, Heinrich-Heine-University, Düsseldorf, Germany; 2 Institute of Biochemistry, Heinrich-Heine-University, Düsseldorf, Germany; 3 Institute of Virology, Heinrich-Heine-University, Düsseldorf, Germany; 4 Institute of Organic Chemistry, Justus Liebig University Giessen, Giessen, Germany; 5 German Primate Center, Leibniz Institute for Primate Research, Göttingen, Germany; 6 Center for Bioinformatics, Saarland University, Saarbrücken, Germany; 7 Medical Faculty and University Hospital Düsseldorf, Institute of Biochemistry and Molecular Biology II, Heinrich-Heine-University, Düsseldorf, Germany; 8 Department of Medicinal Chemistry, School of Pharmacy, University of Washington, Seattle, Washington, United States of America; 9 Department of Pediatrics III, University Children’s Hospital Essen, University Duisburg-Essen, Essen, Germany; University at Buffalo - The State University of New York, UNITED STATES OF AMERICA

## Abstract

A hallmark of the highly conserved CYP4B1 enzyme in mammals is the capability to bioactivate both xenobiotic and endobiotic substrates. However, due to a single amino acid change (p.P427S) within the evolutionary conserved meander region no catalytic activity of the native human CYP4B1 has been identified so far. To identify at which point in human evolution the loss of CYP4B1 activity had occurred, we evaluated the activities of CYP4B1 orthologs from 14 primate genera against 4-ipomeanol and perilla ketone in human liver cells. The activity of recombinant CYP4B1 proteins isolated from *E. coli* was also tested against 4-ipomeanol and lauric acid. Surprisingly, CYP4B1 already became catalytically inactive at the split between apes and monkeys; all tested CYP4B1 orthologs from monkeys were able to bioactivate both protoxins and to hydroxylate lauric acid. Amino acid analysis of the CYP4B1 orthologs revealed four additional evolutionary changes, each affecting the function of ape and human enzymes: p.V71G specific for Denisovans, p.R106C, p.R244H, and an exon deletion found only in the gorilla CYP4B1. Systematic functional analyses proved the negative impact of the genetic changes on CYP4B1 activity and showed that reversion of the mutations restored enzyme activity. The occurrence of five independent inactivating genetic changes in the same gene of closely related species is a clear indication of the importance of inactivating CYP4B1 in apes and humans. Elucidating the evolutionary trigger(s) for CYP4B1 inactivation in our ancestors will ultimately improve our understanding of primate evolution.

## Author summary

Cytochrome P450 monooxygenases are evolutionary old enzymes that have critical roles in developmental processes and in mammals are crucial in maintaining health by metabolizing endogenous and exogenous compounds. The human CYP4B1 enzyme is an enigmatic member of the CYP4 family, developed following the formation of steroid synthesis genes. While the human CYP4B1 enzyme is still expressed but completely inactive, CYP4B1 enzymes of other mammals differ in one critical amino acid in the meander region and were therefore believed to be active. Here, we provide genetic and functional proof that a yet unidentified strong evolutionary selection process also completely inactivated the CYP4B1 orthologs of great apes and gibbons, while all monkeys tested express CYP4B1 enzymes with high activity against hallmark substrates. We also identify specific genetic mutations that occurred independently in the same gene in related species, proofing the importance of inactivating CYP4B1 for hominoid evolution.

## Introduction

Cytochrome P450 (CYP) monooxygenases are heme-thiolate enzymes distributed across all kingdoms of life including animals, plants, bacteria, and viruses [1,2]. In humans, this superfamily comprises 18 families with 57 functional genes, and 58 pseudogenes identified to date [3,4]. The human *CYP* gene battery is largely conserved during evolution across diverse phyla ranging from fish to mammals [5]. To facilitate comparison, the nomenclature system of CYPs is based on evolutionary relationships and on sequence identity. CYPs sharing a minimum of 40% sequence identity are members of the same family, and an identity of more than 55% permits grouping in the same subfamily [6,7].

The CYP4 family is one of the most ancient CYP families and is divided into 72 subfamilies, predominantly present in invertebrates [1,8]. Only seven subfamilies (CYP4A, CYP4B, CYP4F, CYP4T, CYP4V, CYP4X, and CYP4Z) are present in vertebrates [9,10], three (CYP4A, CYP4X, and CYP4Z) are found only in mammals, and CYP4Z is even specific for humans [11,12]. The human *CYP4* genes of subfamilies A, B, X, and Z are clustered on chromosome 1, the six *CYP4F* genes, encoding seven isoforms, are located on chromosome 19, and the *CYP4V2* gene is located on chromosome 4 [9,13]. CYP4 enzymes characteristically ω-hydroxylate fatty acids of variable length, with the chain-length selectivity differing within the six human subfamilies. CYP4F enzymes preferentially metabolize long-chain (C18-C26) fatty acids, CYP4A and CYP4V proteins metabolize medium-chain (C10-C16) fatty acids, and CYP4B was shown to hydroxylate short- to medium-chain (C7-C15) chain fatty acids [11,14,15]. Besides their involvement in endobiotic metabolism, enzymes of the CYP4 family also participate in the metabolism of xenobiotics, but compared to the CYP1-3 families they are less dominant in the metabolism of exogenous compounds [13,16].

Among all members of the CYP4 family, CYP4B1 is primary involved in the metabolism of certain xenobiotics and, therefore, has been studied most extensively for its role in bioactivation of protoxic compounds to reactive products, exerting toxicological effects via protein/DNA binding [17]. Beyond the ω-hydroxylation activity of CYP4B1 towards endogenous fatty acids, rabbit CYP4B1 was later described to also hydroxylate the branched, medium-chain fatty acid anticonvulsant valproic acid (VPA) [18,19]. As a primary extrahepatic enzyme with the highest expression found in the respiratory system [20,21], CYP4B1 has been studied for its ability to metabolize compounds that elicit organ-specific toxicities. Thus, CYP4B1 has been shown to participate substantially in the bioactivation of pneumotoxins including 3-methylindole (3-MI) and 4-ipomeanol (4-IPO) [17,22,23].

4-IPO, a hallmark substrate of CYP4B1, naturally occurring in fungus-infected sweet potatoes (*Ipomoea batata*) causes severe pulmonary toxicities, initially observed in livestock and later confirmed in animal studies in the laboratory [24–27]. Among the different CYP4B1 orthologs, the rabbit enzyme was most commonly used for 4-IPO activity studies as it is a highly active protein that can be easily isolated from the lungs of the animals [17]. Importantly, characterization of CYP4B1 ortholog activities revealed species-specific differences in CYP4B1 activities between humans and experimental animals. Despite human and rabbit CYP4B1 sharing ~ 84% sequence identity, the human enzyme is unable to bioactivate 4-IPO or other characteristic CYP4B1 substrates [28,29]. This functional inactivity of human CYP4B1 has been attributed to a single amino acid exchange, p.P427S, in the evolutionary conserved meander region [30], hypothesized to disrupt the ordered framework of the structure imposed by the ERR triad, thus causing a defective heme incorporation. Re-introducing the proline at position 427 in the human enzyme, p.S427P, restored the catalytic activity of human CYP4B1 [30–32], and substantially improved the protein stability of the human enzyme [28].

In the present study, we sought to identify the critical time point(s) in the evolution of *Homo sapiens* at which changes in the *CYP4B1* gene occurred, thereby influencing the activity of the enzyme against a panel of representative protoxic and non-protoxic compounds. Sequence comparison and functional analyses of CYP4B1 orthologs from prosimians, Old World monkeys (OWMs), and hominoids (apes and humans), revealed a complete loss of CYP4B1 enzyme activity already at the evolutionary level of gibbons and great apes. Most striking, in addition to the p.P427S alteration specific to the human clade, we identified three additional hot spots where amino acids in the CYP4B1 sequence strongly correlate with the enzymatic activity. By systematically introducing these amino acid changes either singly or in combinations into the well-characterized rabbit and human *CYP4B1* cDNAs, and by analyzing the activities of these mutated enzymes in human liver cells against specific protoxic substrates, we unequivocally demonstrated that distinct genetic inactivation in the *CYP4B1* gene occurred independently in all hominoids. Thus, the inactivation of CYP4B1 must have been important in the evolution of *Homo sapiens*.

## Results

### Assessment and evaluation of primate CYP4B1 orthologs

**Fig 1:**
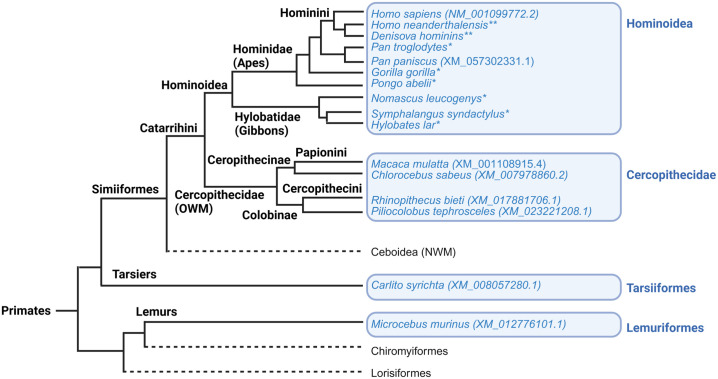
Schematic primate phylogeny based on whole-genome sequences of primates (compiled from [37–39]). CYP4B1 sequences from species marked in blue were used in this study for CYP4B1 characterization. Sequences were either downloaded from NCBI, provided by the MPI, Leipzig (**), or curated by our team (*) after sequencing of RNA extracted from cryopreserved lung tissues.

To shed light on evolutionary changes in CYP4B1, we first visualized the phylogenetic tree of primates used in our study (Fig 1) [33,34]. Based on public databases (NCBI, Ensembl), we obtained the amino acid sequences of CYP4B1 from a tarsier (*Carlito syrichta*/Philippine tarsier XM008057280.1), a lemur (*Microcebus murinus*/gray mouse lemur XM_012776101.1), and four OWMs (*Rhinopithecus bieti*/Yunnan snub-nosed monkey XM_017881706.1; *Piliocolobus tephrosceles*/Ugandan red colobus XM_023221208.1; *Chlorocebus sabaeus*/African green monkey XM007978860.2 and *Macaca mulatta*/rhesus monkey XM_001108915.4). We also extracted the sequence information for *Homo sapiens*/modern human (NM_00131916.1), *Pan paniscus*/bonobo (XM_057302331.1), and *Oryctolagus cuniculus*/rabbit (NM_001082103) enzymes. In addition, Prof. Dr. Svante Pääbo from the Max Planck Institute (MPI) for Evolutionary Anthropology, University of Leipzig, provided us with the *CYP4B1* DNA sequences of Denisovan and Altai Neanderthal individuals, two sibling lineages of *Homo sapiens* [35,36].

While the CYP4B1 data of monkeys, bonobo, *Homo sapiens* and rabbit were well annotated, only low quality CYP4B1 transcript sequences were available for *Pongo abelii*/North Sumatran orangutan (XM_054535134.2) and *Gorilla gorilla gorilla*/Western lowland gorilla (Ensembl, ENSGGOT00000016915.3), which partly included premature stop codons (orangutan) or wrongly annotated splicing (gorilla). Surprisingly, no CYP4B1 genetic information was available for gibbons. The *Pan troglodytes*/chimpanzee amino acid sequence (Ensembl, ENSPTRT00000100736_1) differed at amino acid position 106 (Arg versus Cys) from those of other great apes. To correct these inconsistencies, we extracted mRNAs from cryoconserved lung tissues of three gorillas, four chimpanzees, four orangutans, and five gibbons (including four species). Upon reverse transcription, cDNA sequencing revealed high degrees of sequence identity (more than 95%) of Hominoidea CYP4B1 sequences to the human CYP4B1 protein (S1 File) and confirmed that the *CYP4B1* gene also exists in gibbons.

**Fig 2:**
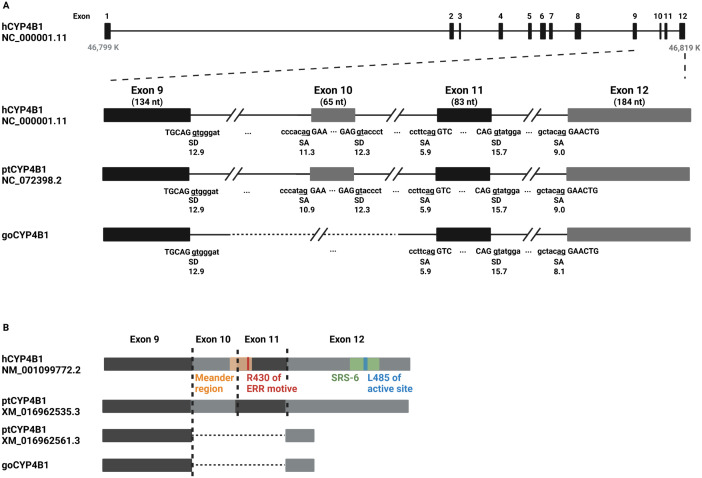
Sequence analysis of gorilla CYP4B1. **(A)** The endogenous *Homo sapiens* CYP4B1 (hCYP4B1) located on human chromosome 1 (NC_000001.11 NCBI ID) encodes 12 exons. **(B)** gDNA sequence analysis of three gorilla lung tissues samples revealed a gap spanning part of intron 9, exon 10, and intron 10 when compared to hCYP4B1 and chimpanzee CYP4B1 (ptCYP4B1, NC_072398.2 NCBI-ID). The strengths/scores of the splice acceptor (SA) and splice donor (SD) sites were calculated using MAXENT or HBS splice site algorithms. At the mRNA level, gorilla CYP4B1 (goCYP4B1) is lacking not only exon 10, but also exon 11 as shown by mRNA sequencing of lung tissues. Functional domains highlighted as follows: substrate recognition sites (green), heme covalent binding site (yellow), the ERR triad (red), active site (blue), and meander region (orange).

The gorilla CYP4B1 ortholog is unique since the exon 10 sequence present in human and other great apes (e.g., the chimpanzee) was missing in all six alleles of the three gorillas analyzed due to deletions in the surrounding introns (Fig 2A). In addition, a closer look at the gorilla CYP4B1 transcript revealed that the gorilla exon 10 (corresponding to exon 11 in human and chimpanzee) is also not included (Fig 2B). Analysis of the splice donor and acceptor sites revealed a weak splice acceptor in the 5’ part of gorilla exon 10 (corresponding exon 11 in human and chimpanzee) thus, this exon is largely skipped in the transcript leading to a frameshift in the last exon that codes for a protein of 410 amino acids (S1 Text).

### Enzyme activities of different CYP4B1 orthologs against key substrates

Next, we evaluated the enzyme activities of the 14 different CYP4B1 orthologs in two experimental set ups: (i) by stable expression of the proteins in two human liver cancer-derived cell lines and analysis of their efficiency of processing the two protoxins 4-IPO and the structurally related perilla ketone (PK), and (ii) by testing the recombinantly expressed CYP4B1 proteins for conversion of lauric acid (LA) and 4-IPO as representative CYP4B1 substrates.

**Fig 3:**
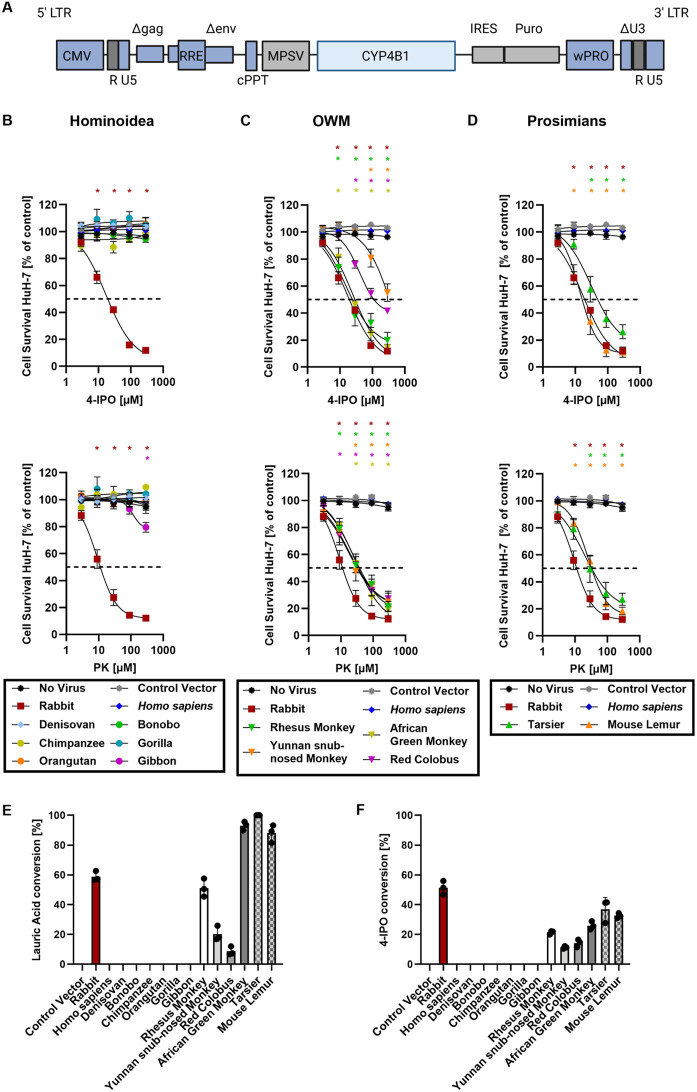
Functional analysis of primate CYP4B1 orthologs. **(A)** The cDNAs of CYP4B1 enzymes from different primate clades, including prosimians, Old World monkeys (OWMs), gibbon, great apes, and humans were cloned into a lentiviral expressing vector upstream of an IRES-Puro site. CMV: CMV promoter; LTR: long terminal repeat; RRE: Rev responsive element, cPPT: central polypurine binding tract; MPSV: U3 promoter of the myeloproliferative sarcoma virus; IRES: internal ribosomal entry site; Puro: cDNA for puromycin resistance; WPRO: woodchuck hepatitis virus post-transcriptional regulatory element optimized. **(B-D)** The activities of CYP4B1 orthologs in HuH-7 liver cells were evaluated in MTS assays following a 24 hrs exposure to either 4-ipomeanol (4-IPO, upper graphs) or Perilla ketone (PK, lower graphs). Experiments were performed with a minimum of three individual replicates each. The graphs are divided based on primate lineages: gibbon and great apes **(B)**, Old World monkeys **(C)**, and prosimians **(D)**. **(E-F)** Specific activities for hydroxylation of LA and metabolization 4-IPO by recombinant CYP4B1 orthologs isolated from *E. coli*. For each data set at least three individual replicates were measured and are shown as mean ± SEM. For statistical analysis, a multiple comparison one-way ANOVA with subsequent Dunnett’s-Post-hoc test was used to determine significant differences between measuring points compared to untreated controls: p-values < 0.05 were defined as significant and were marked with an asterix (*). The underlying data for the graphs in this figure can be found in S1 Data. For calculation of the half-maximal effective concentration that reduces the surviving cell number by 50% (GI_50_ values), a non-linear fit model was applied to each data set (S3 Data).

To this end, we first cloned the cDNAs of the 14 CYP4B1 orthologs into the lentiviral IRES-Puro expression vector shown in Fig 3A, produced and harvested recombinant lentiviral particles and then transduced two human liver cell lines, HepG2 and HuH-7, as previously described [28,40,41]. The transduced cells were selected for five days with puromycin (until all non-transduced cells had died), seeded in 96-well plates and then exposed to increasing concentrations of 4-IPO or PK (2.9, 9, 29, 90, 290 µM). After 24 hours, the mitochondrial activities of the surviving cells were determined by standard MTS assay and the half-maximal effective concentrations for 50% activity (GI_50_ values) were calculated.

As expected, the CYP4B1 orthologs of *Homo sapiens* and Denisovan, both carrying the inactivating p.P427S alteration, were not able to activate 4-IPO or PK in neither HuH-7 (Fig 3B) nor HepG2 cells (S1 Fig), whereas the rabbit CYP4B1 analyzed in parallel in the same cell lines was highly active with Gl_50_ of 20.3 and 12.9 µM for 4-IPO and PK in HuH-7 cells, respectively (S3 Data). Using HepG2 cells for expressing the CYP4B1 enzymes showed the same trends, albeit the toxicities were not as profound (S1 Fig). Surprisingly, the CYP4B1 orthologs of neither great apes nor gibbon exhibited any enzymatic activity when exposed to 4-IPO or PK in HuH-7 or HepG2 cells (Figs 3B and S1). In contrast, all CYP4B1 enzymes of the four OWMs and the two prosimian species exhibited high metabolic activities towards 4-IPO and PK in HuH-7, and to a lesser degree, in HepG2 cells (Figs 3C-D and S1).

In order to investigate whether an artificial full-length gorilla CYP4B1 might still be active, we compensated for the genomic exon deletion in the gorilla genome by including the human exon 10 sequence followed by the gorilla exon 10 and 11, thus creating a chimeric CYP4B1 enzyme with 98% identity compared to the *Homo sapiens* wild type protein (S2 Fig). This artificial protein with p.P427 encoded by the gorilla exon 11 was expressed in HuH-7 cells that subsequently were exposed to increasing concentrations of 4-IPO and PK. Neither the wild type nor the chimeric gorilla CYP4B1 were able to bioactivate one of the tested protoxins, suggesting that the exonic deletion in the gorilla genome is not the crucial factor for inactivation of CYP4B1, but rather seems to be due to a genetic drift, since no other CYP4B1 orthologs from great apes have similar mutations and are still non-functional (S2 Fig).

Finally, as well characterized purified CYP4B1 orthologs of the rabbit and other species (except *Homo sapiens*) are known to hydroxylate LA as a representative medium-chain fatty acid [42,31], we also set up to express the mammalian CYP4B1 cDNAs in *E. coli*. To this end, we deleted the N-terminal membrane anchors to achieve soluble expression. The recombinant enzymes were isolated and their concentrations were determined by recording CO-difference spectra (S3 Fig). As the CYP4B1 orthologs of apes and humans did not display any peaks at 450 nm in the CO-difference spectra, we used SDS-PAGE and western blotting to verify their soluble expression (S3 Fig). Analysis of the catalytic activities of the CYP4B1 orthologs towards LA and 4-IPO resulted in highly similar activity patterns as observed in the cell culture-based experimental system. Only the CYP4B1 orthologs from OWMs and prosimians as well as the rabbit enzyme were capable of hydroxylating LA and metabolizing 4-IPO, whereas the CYP4B1 orthologs from apes and humans were completely inactive (Fig 3E and 3F).

In summary, these results suggest that a complete loss of activity of the CYP4B1 enzyme had occurred in all great apes and humans, clearly separating them from OWMs and more distantly related primates.

### Expression and localization of the CYP4B1 orthologs in cell lines

**Fig 4:**
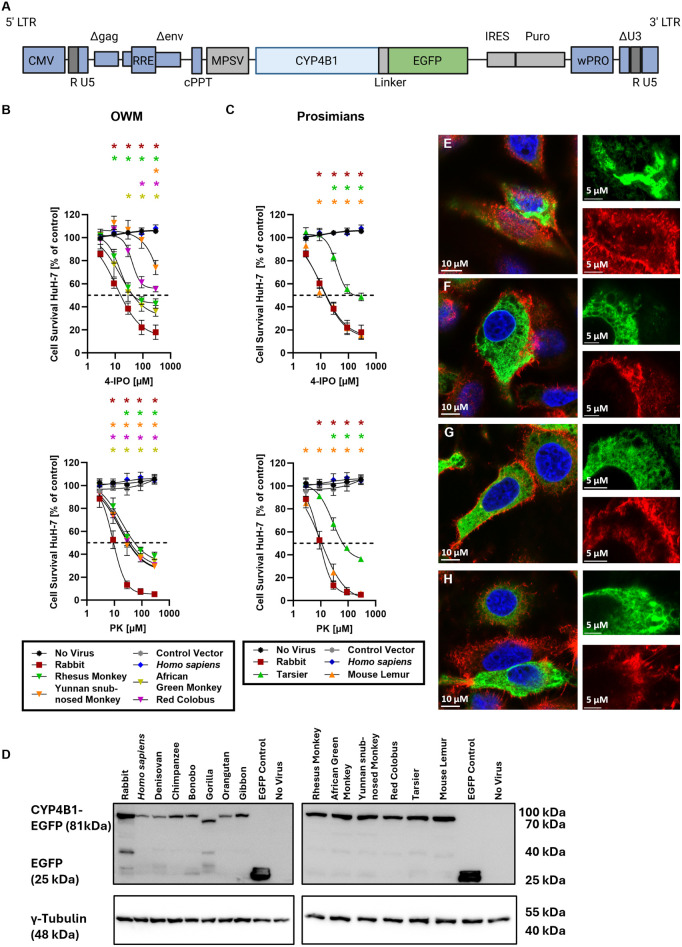
Activity, expression and localization of CYP4B1-EGFP fusion proteins in HuH-7 cells. **(A)** Design of the lentiviral vector used for overexpression of the CYP4B1-EGFP fusion proteins. **(B-C)** Enzymatic activities and of the fusion proteins of Old World monkeys (OWMs) and prosimians against 4-IPO and PK in HuH-7 cells. GI_50_ values calculated based on the non-linear cur fit model can be found in S3 Data. **(D)** Expression levels of CYP4B1-EGFP fusion proteins in polyclonal HuH-7 cell cultures were estimated by western blot. For detection, an anti-EGFP antibody was used; γ-Tubulin was used as a loading control. **(E-H)** Subcellular localization of the fusion proteins of OWMs and prosimians. Shown are representative images of cells overexpressing either rabbit **(E)**, mouse lemur **(F)**, bonobo **(G)**, or wild type *Homo sapiens* **(H)** CYP4B1. The cells were co-stained for nuclei (HOECHST) and F-actin (Phalloidin-TRITC). 10 µM or 5 µM scale bars are implemented on overview images or detailed images showing only one fluorescent channel (EGFP or phalloidin), respectively. For each data set at least three individual replicates were measured and are shown as mean ± SEM. For statistical analysis, a multiple comparison one-way ANOVA with subsequent Dunnett’s-Post-hoc test was used to determine significant differences between measuring points compared to untreated controls: p-values < 0.05 were defined as significant and were marked with an asterix (*). The underlying data for the graphs in this figure can be found in S1 Data.

Next, we wanted to exclude the possibility that the inactivity of CYP4B1 orthologs from apes and humans was neither due to incorrect expression in the endoplasmic reticulum (ER) of cells nor to decreased expression levels that could occur as a consequence of protein instability. As no antibody was available that can equally bind all different CYP4B1 orthologs, we generated CYP4B1-EGFP fusion proteins (Fig 4A), as previously published [28]. To identify any effect of the EGFP fusion on catalytic activity of the enzymes, we first expressed the EPFG-tagged CYP4B1 enzymes in HuH-7 cells, using the lentiviral expression vector shown in Fig 4A. Exposure of the puromycin-resistant cells to increasing concentrations of 4-IPO or PK showed a slight diminished activity of the OWM and the prosimian EGFP-fusion enzymes in activating the prodrugs. However, the fusion proteins were still capable of processing both protoxins (Fig 4B and 4C and S3 Data). Western blot analysis (Fig 4D) of whole cell lysates for all orthologs demonstrated some variations in the protein expression levels. The expression levels of CYP4B1-EGFP fused orthologs from OWMs and prosimians with the higher catalytic activity also showed higher signal intensities, similar to that of rabbit CYP4B1. The expression levels of the *Homo sapiens* and Denisovan enzymes were decreased compared to the rabbit protein, probably due to the influence of the p.P427S alteration and its adverse effect on the half-life of the protein as demonstrated previously [28]. Signal intensities of CYP4B1-EGFP fusion proteins of the gibbon and great apes were only slightly decreased when compared to the rabbit and the human enzymes.

Finally, the localization of the EGFP-tagged CYP4B1 orthologs in HuH-7 cells was analyzed by fluorescence microscopy. To this end, the F-actin of cells stably expressing the fusion enzymes was stained with Phalloidin-TRITC (red) and the nuclei was stained with Hoechst (blue). The representative microscopic images shown in Fig 4E-H revealed that the CYP4B1 fusion proteins of the rabbit, the mouse lemur, the bonobo, and the *Homo sapiens* (p.P427S) were all correctly located in the endoplasmic reticulum (ER) of the cells, independently of whether the enzymes were capable of processing 4-IPO/PK or not.

### Identification of potential evolutionary hot spots influencing CYP4B1 catalytic activity

Since we demonstrated that CYP4B1 orthologs from apes were incapable of metabolizing 4-IPO, PK, and LA despite having a proline at position 427 (except the gorilla CYP4B1 gene having the exon deletion) in the conserved meander region, we hypothesized that at least one additional change in their amino acid sequence must have occurred that led to catalytic inactivation. Thus, the CYP4B1 amino acid sequences were systematically analyzed to identify evolutionary adaptations, possibly responsible for the adverse catalytical function.

**Fig 5:**
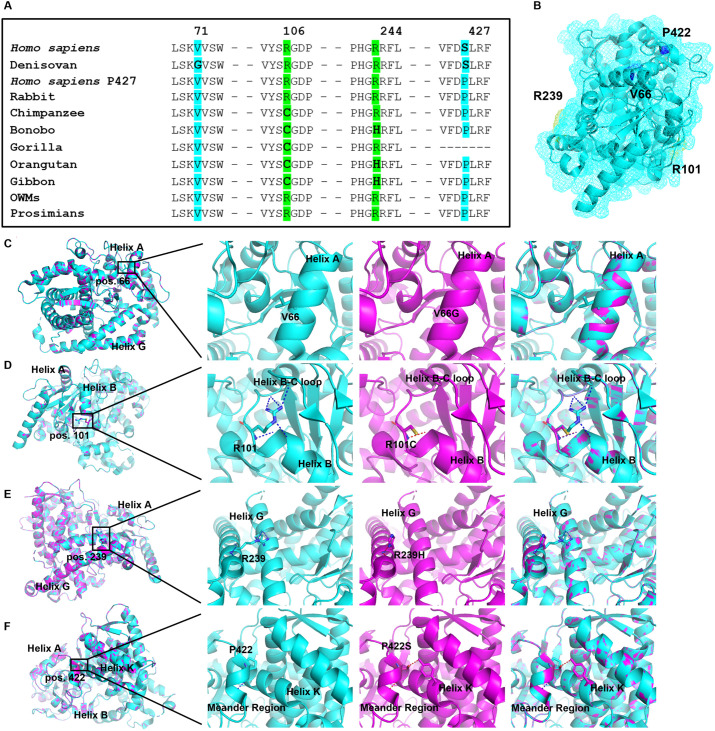
*In silico* modeling of potential key amino acid positions for the functional activity of mammalian CYP4B1. **(A)** Multiplexed comparison of CYP4B1 amino acid sequences to identify hot spots with potential impact on catalytic function. Human-based amino acid exchanges are colored in blue, while substitutions in apes are colored in green. **(B)** The location of all corresponding residues is depicted within the rabbit CYP4B1 model, PDB: 5T6Q (rabbit CYP4B1 complexed with octane). Within the rabbit CYP4B1, the amino acid sequence positions are shifted by -5 when compared to primate CYP4B1 sequences. Residues are colored in blue (p.V66 and p.P422) or green (p.R101 and p.R239). **(C-F)** Side directed mutagenesis of all four key amino acid positions (p.V66, p.R101, p.R239, and p.P422) was performed based on the rabbit CYP4B1 protein structure. Overall structure is colored in cyan (WT sequence) or magenta (mutated enzyme), amino acid residues are colored by atoms, and polar interactions are depicted as yellow dots. From left to right: location within the whole enzyme, defined WT amino acid sequence of rabbit CYP4B1, defined mutated amino acid, superimposition of WT and mutated enzyme.

First, multiplex amino acid alignments were analyzed with focus on residues differing between catalytically active and inactive orthologs. Sequence comparison of three CYP4B1 orthologs of the humans (*Homo sapiens*, Neanderthals and Denisovan) revealed identical amino acid sequences except for a single amino acid exchange of an evolutionary conserved valine by glycine (p.V71G) [43,44] that was restricted to Denisovans. A substitution of the amino acid at this position to glycine could not be identified in CYP4B1 sequences from other species or in other *Homo sapiens* CYP-enzymes belonging to family 4 (Figs 5A and S4 and S1 File) and is therefore unique for the Denisovan CYP4B1 ortholog.

Comparison of CYP4B1 amino acid sequences from apes and humans revealed additional changes at the positions 106 and 244 (referred to the *Homo sapiens* CYP4B1 ortholog) in the helix B-C loop or helix G, respectively [43]. Whereas catalytically active orthologs from different species, e.g., from rabbit, monkeys, cattle, mice, and also the *Homo sapiens* (wild type p.S427 and re-activated p.S427P enzyme), have an arginine at position 106 (Figs 5A and S4), the inactive orthologs from apes have a cysteine residue (C106) instead. At position 244, an arginine is present in all active CYP4B1 orthologs from various species including hamster, rabbit, horse, mouse, cattle, and monkeys as well as *Homo sapiens*, and surprisingly also in the chimpanzee and gorilla enzymes (Figs 5A and S4 and S1 File). However, the CYP4B1 sequences from bonobo, orangutan, and gibbon showed an arginine-to-histidine exchange at this position (p.R244H) (Fig 5A). Thus, both amino acids in positions 106 and 244 seem to be evolutionarily conserved in active enzymes and thus may be important for CYP4B1 catalytic functions.

To visualize the consequences of the missense mutations p.V71G, p.R106C, and p.R244H *in silico* in a functional CYP4B1 enzyme, we utilized the 3D-structure of rabbit CYP4B1 (PDB 5T6Q) that was crystalized in complex with octane [43]. Whilst introduction of the valine-to-glycine substitution at position 71 (corresponding to amino acid 66 in rabbit CYP4B1) seems to affect neither protein conformation nor polar interactions, this substitution still preserves the characteristics of a small non-polar side chain, thus suggesting that this alteration might not have significant effects on the protein activity (Fig 5C). In contrast, introduction of substitutions p.R106C (p.R101C in rabbit CYP4B1) and p.R244H (p.R239H in rabbit CYP4B1) seem to have a more profound impact on the CYP4B1 intrinsic polar interactions: p.R101C causes a switch from an alkaline amino acid to a short polar one, thus influencing the amount of polar interactions at the side of the helix B-C loop which is part of the substrate access channel and associated to the ER membrane [45] (Fig 5D). p.R239H mutation preserves the alkaline character of the residue, however, changes the conformation of the side chain from linear into a more complex pentene ring system. Here, the conformational changes influence the orientation of the side chain within the protein structure leading to a clear shift in H-bonds present (Fig 5E).

### Influence of p.V71G, p.R106C, and p.R244H on CYP4B1-mediated cytotoxicity and expression

To systematically investigate potential influences of each identified hot spot on CYP4B1 activity, the mutations p.V71G, p.R106C, and p.R244H were introduced by side-directed mutagenesis into active CYP4B1 orthologs.

**Fig 6:**
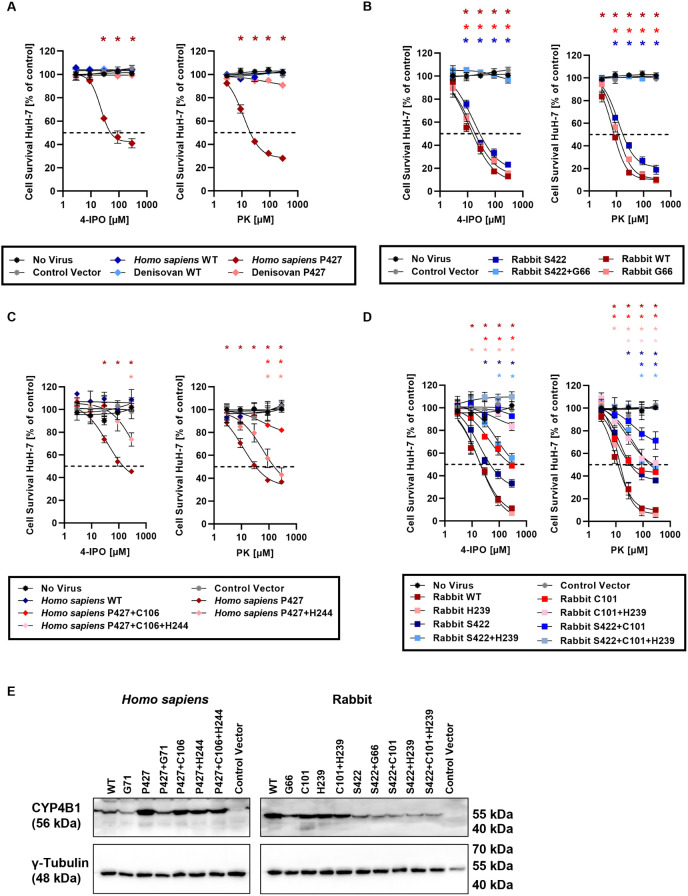
Functional consequences of the four critical amino acid substitutions in Homo sapiens and rabbit CYP4B1. All experiments were performed with a minimum of three individual replicates each in the human liver carcinoma cell line HuH-7. **(A)** Enzyme activity of the Homo sapiens CYP4B1 wild type protein and p.S427P variant and of the Denisovan wild type (S427, G71) and p.S427P variant against increasing concentrations of 4-IPO or PK. **(B)** Enzyme activity of the rabbit CYP4B1 wild type protein and the single or double mutated variants p.P422S and p.V66G. **(C)** Functional effects of the amino acid substitutions p.R106C and p.R244H introduced in the Homo sapiens CYP4B1 P427 enzyme. **(D)**. Functional implications of the amino acid substitutions at the corresponding positions in the rabbit CYP4B1 wild type and p.P422S enzyme. **(A-D)** For each data set at least three individual replicates were measured and are shown as mean ± SEM. For statistical analysis, a multiple comparison one-way ANOVA with subsequent Dunnett’s-Post-hoc test was used to determine significant differences between measuring points compared to untreated controls: p-values < 0.05 were defined as significant and were marked with an asterix (*). The GI_50_ values for the respective Homo sapiens and rabbit enzymes were calculated based on a non-linear curve fit model and can be found in S3 Data. The underlying data for the graphs in this figure can be found in S1 Data. **(E)**. Effect of the amino acid exchanges p.P427S, p.V71G, p.R106C and p.R244H on the expression of Homo sapiens and rabbit (wild type and p.P422S variant) CYP4B1 in HuH-7. Human CYP4B1 enzymes were detected with an anti-human CYP4B1 polyclonal antibody. Rabbit CYP4B1 enzymes were detected with an anti-rabbit CYP4B1 polyclonal antibody. γ-tubulin served as a loading control.

As shown in Figs 6A, 6C, and S5, the *Homo sapiens* wild type CYP4B1 was not activated by introduction of p.V71G, as the survival of HuH-7 and HepG2 cells overexpressing this mutated enzyme was not affected even by highest protoxin concentrations. As well established, the p.S427P substitution restored the activity of the *Homo sapiens* CYP4B1 enzyme with GI_50_ values of 32.5 µM or 104.7 µM for PK or 4-IPO, respectively (S3 Data). Surprisingly, however, introduction of the valine-to-glycine switch found exclusively in the Denisovan human into the re-activated *Homo sapiens* P427 resulted in a complete inactivation of this enzyme. Introduction of p.V66G (p.V71G in all other orthologs) into the highly active rabbit CYP4B1 also had a negative impact on enzyme activity (Figs 6B and S5 and S3 Data); the GI_50_ value for the rabbit G66 enzyme increased from 9.0 µM to 13.7 µM when incubated with PK and from 16.4 µM to 21.6 µM following 4-IPO treatment. Finally, introducing the p.V66G substitution into the rabbit CYP4B1 S422 variant with diminished activity led to a complete loss of the catalytic function in this double-mutant protein (Figs 6B and S5 and S3 Data). These data unequivocally demonstrated the negative effects of this amino acid substitution found exclusively in the Denisovan human.

Introduction of the p.R106C mutation in the re-activated (P427) *Homo sapiens* CYP4B1 led to a complete loss of the catalytic activity against 4-IPO, regardless if this substitution was introduced as a single or double alteration together with p.R244H in HuH-7 or HepG2 cells. In contrast, the single mutation p.R244H only reduced the activity against 4-IPO (GI_50_ = 599.8 µM). Similar effects, although not as profound, were observed following PK exposure (Figs 6D and S5 and S3 Data).

Next, introducing these two amino acid substitutions, p.R101C and p.R239H, into the rabbit CYP4B1 wild type and the rabbit CYP4B1 S422 enzymes expressed in HuH-7 and HepG2 liver cells showed similar results upon exposure to 4-IPO and PK. Whereas the activity of the wild type rabbit CYP4B1 protein was clearly affected by the introduction of both mutations, the activity of the single arginine-to-histidine exchange (p.R239H) was not associated with any activity reduction and the single p.R101C substitution only with 6.1- to 11.0-fold increased GI_50_ values for PK or 4-IPO, respectively. In contrast, the already reduced enzymatic activity of the rabbit CYP4B1 S422 protein was further diminished by both single amino acid substitutions and completely abolished in the triple mutant rabbit CYP4B1 S422 + C101 + H239 protein (Figs 6D and S5 and S3 Data).

Since previous research demonstrated an positive effect on the wild type *Homo sapiens* CYP4B1 protein expression/half-life in cells after introducing the p.S427P exchange [28], potential impacts of the key positional amino acid exchanges in *Homo sapiens* and rabbit CYP4B1 enzymes were analyzed. Therefore, we expressed the cDNAs at similar multiplicity of infection (MOI) in HuH-7 cells and analyzed the puromycin resistant cells via immunoblotting with either an α-human CYP4B1 or an α-rabbit CYP4B1 antibody. As shown in Fig 6E, the p.P427S and p.V71G substitutions clearly affected the expression of the protein in HuH-7 cells, while the signal intensities for the p.R106C and p.R244H mutated proteins were comparable to the initial *Homo sapiens* CYP4B1 P427 or rabbit CYP4B1 wild type enzymes. We confirmed these findings by determining the reduced proteins half-life for the proline-to-serine and the valine-to-glycine substitutions in our established flow cytometry-based assay [28] (S6 Fig).

### Restoring the activity of gibbon and great ape CYP4B1 orthologs

Finally, in order to prove that the alterations p.C106R and p.H244R were ultimately responsible for the inactivation of CYP4B1 in apes, we re-introduced an arginine at these two positions in the CYP4B1 orthologs from bonobo, orangutan, and gibbon and then tested the enzymatic activity of the variant proteins against 4-IPO or PK.

**Fig 7:**
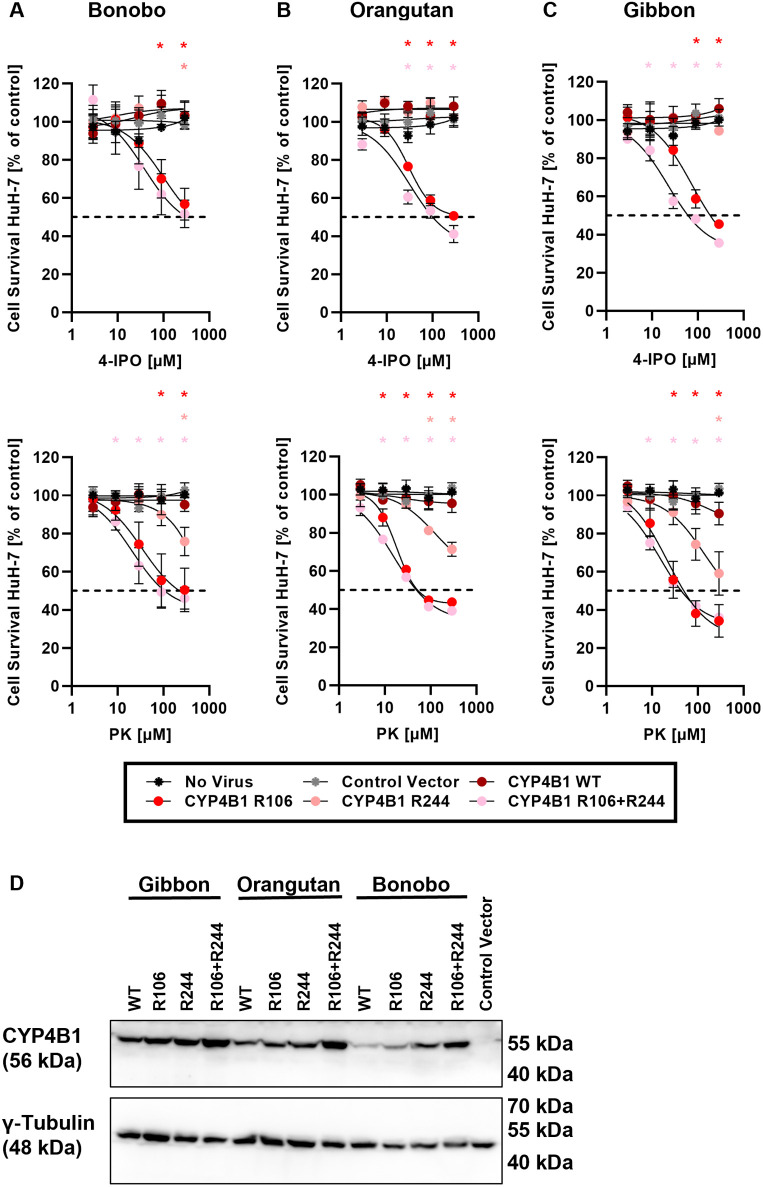
Restoring the activity of gibbon and two great ape CYP4B1 orthologs by p.C106R and p.H244R substitution. The enzymatic activity of hominoid CYP4B1 proteins was evaluated in HuH-7 cells after a 24h exposure to either 4-IPO or PK. **(A)** Enzymatic activity of bonobo CYP4B1 variants. **(B)** Catalytic activity of orangutan CYP4B1 variants. **(C)** Enzyme activity of gibbon CYP4B1 variants. For each data set at least three individual replicates were measured and are shown as mean ± SEM. For statistical analysis, a multiple comparison one-way ANOVA with subsequent Dunnett’s-Post-hoc test was used to determine significant differences between measuring points compared to untreated controls: p-values < 0.05 were defined as significant and were marked with an asterix (*). GI_50_ values calculated based on a non-linear curve fit model (S3 Data). The underlying data for the graphs in this figure can be found in S1 Data. **(D)**. Effect of the amino acid exchanges p.C106R and p.H244R on expression levels of gibbon, orangutan, and bonobo CYP4B1 proteins in HuH-7 cells. The hominoid proteins were detected with an anti-human CYP4B1 antibody. γ-tubulin served as a loading control.

As shown in Figs 7A-C and S5, all three CYP4B1 orthologs carrying the single p.C106R mutation exhibited *de novo* catalytic activity against both protoxins when expressed in HuH-7 and HepG2 cells. The strongest impact of this substitution was noted for the gibbon CYP4B1 with GI_50_ values of 191.4 µM or 63.7 µM for 4-IPO or PK, respectively (S3 Data). In contrast, the histidine-to-arginine switch at position 244 (p.H244R) did not restore the catalytic activity of the enzymes towards 4-IPO, while metabolization of PK was observed at higher concentrations. However, the re-introduction of the double mutations (p.C106R + p.H244R) further increased the turnover of 4-IPO, while the effects on PK processing were not as obvious. Although no profound adverse effects were noted in the expression of *Homo sapiens* or rabbit CYP4B1 in HuH-7 cells by western blotting (Fig 6G), analyzing the protein expression levels of the three hominoid CYP4B1s in HuH-7 cells suggested at least slight improvements in protein stability with the arginine exchanges (Fig 7D).

Finally, we conducted a Fixed Effects Likelihood (FEL) analysis [46] to assess whether the four hot spots, which we have demonstrated to be critical for enzymatic activity of CYP4B1, are subject to relaxed selection. Codon-wise comparison of non-synonymous (dN) and synonymous (dS) substitution rates revealed 118 sites under purifying selection. However, for the hot spot codons 71, 106, 244, and 427, the FEL analysis yielded ω (dN/dS) values close to one at a significance threshold of p = 0.1, suggesting a regime of relaxed selection at these positions. Overall, the FEL analysis of *CYP4B1* demonstrated that the gene has evolved under neutral selection, suggesting that dietary factors have not exerted significant evolutionary pressure in primates. Nonetheless, the observation that nearly one quarter of all *CYP4B1* codons exhibit significantly different ω values across distinct branches of the phylogenetic tree leaves open the possibility of lineage-specific purifying selection, potentially reflecting the metabolic role of the gene (S4 Data).

## Discussion

In mammals, the predominantly extrahepatic CYP4B1 shares with other members of the CYP4 family the capacity to ω-hydroxylate medium-chain fatty acids, but seems to be primarily involved in the metabolism of protoxic xenobiotics, including 4-IPO, PK, valproic acid and numerous aromatic amines [17]. It is also well established that the *Homo sapiens* CYP4B1 enzyme is catalytically inactive due to a single amino acid alteration (p.P427S) in the evolutionary conserved meander region. Importantly, reintroducing the proline at position 427 re-activates the *Homo sapiens* CYP4B1 with similar substrate specificity compared to the rabbit CYP4B1, which is the most commonly studied CYP4B1 to date. As all known CYP4B1 orthologs carry a proline at the corresponding position in the meander region, it was so far commonly believed that CYP4B1 turned inactive with the divergence of the *Homo* and *Pan* clades [32].

In line with this assumption are early genomic analyses of modern human populations identifying several genetic variants that completely abolish any catalytic activity of the *Homo sapiens* CYP4B1 [47,48]. In the white French population, three allelic variants were detected that encoded missense mutations with premature stop codons: p.R173W (CYP1*3), p.S322G (CYP1*4), and p.M331I (CYP1*5). A fourth allelic variant, CYP1*2, even harbors three missense mutations (p.M331I, p.R340C, and p.R375C) and a double-nucleotide deletion (c.AT881–882del), thus exemplifying evolutionary accumulation of mutational hits in a locus of a nonfunctional gene [47]. In addition, genomic studies in the Japanese population also revealed the occurrence of these CYP4B1 alleles, and uncovered two other allelic variants: CYP1*6 (p.R173W and p.V345I) and CYP1*7 that shows the same double nucleotide deletion as CYP1*5 but only the first two missense mutations (p.M331I and p.R340C) [48]. Here, the presence of several at least highly similar genomic alterations in two more distant human populations emphasizes that the mutational hits must have occurred early in human evolution and did not promote a selective disadvantage. High-throughput next generation sequencing revealed that the *Homo sapiens CYP4B1* gene carries more than 1,000 SNPs located within exonic regions [23] and that the presence of specific SNPs was even associated with an increased risk for lung and gastric cancer [49,50] as well as breast cancer [51]. Importantly, all single amino acid substitutions functionally characterized within this work are also listed as SNPs occurring in the CYP4B1 alleles of *Homo sapiens*: rs2148404363 (p.V71G), rs138753850 (p.R106C), and rs139993247 (p.R244H). However, these SNPs are rare (rs138753850, C/T, 30/264690, TOPMED; rs139993247, G/A, 73/264690, TOPMED; rs2148404363, T/G, no frequency stated). Most intriguingly however, is the fact that the re-activating SNP, p.S427P, was never detected in any human sample, emphasizing again that an inactivated CYP4B1 enzyme must still be of importance for *Homo sapiens*.

The fact that a serine is also found at position 427 in CYP4B1 in archaic Neanderthals and Denisovans still fits the idea of this p.P427S substitutions being a ‘truly human-specific’ step in evolution. However, a note of caution seems appropriate as high-quality genome data only exists for a few individuals of archaic humans (https://www.eva.mpg.de/genetics/genome-projects/) and as multiple other human groups (*H. florensiensis*, *H. heidelbergensis*, *H. erectus*, or *Australopithecus*) have been identified [52], for whom no genome data are available [38,53,54]. In contrast to Neanderthals, for whom an extensive fossil record and multiple high-quality genomes are available, genomic information for Denisovans remains limited to a single high-coverage genome from a fossil found in the Denisova Cave, Siberia [36]. However, additional Denisovan-like fossils discovered in distant regions, e.g., Tibet and Laos, provided valuable insights into related archaic lineages that interbred with modern humans. These sister lineages exhibit varying degrees of genetic divergence from the original Siberian Denisovan, likely reflecting the Denisovans’ broad geographic distribution [36]. As a result, it remains uncertain whether the Denisovan-specific amino acid substitution identified in our study was universally present across all Denisovan populations. Nevertheless, our findings that the CYP4B1 enzymes of the great apes and the gibbons are also non-functional against the hallmark protoxins (4-IPO and PK) and also lauric acid, clearly indicate a much earlier point in hominoid evolution where the activity of the CYP4B1 protein became problematic for further development. Here, the fact that CYP4B1 from great apes all carry the evolutionary conserved proline at 427 is highly deceiving, as there are multiple ways to inactivate the function of this enzyme. Due to the availability of an increasing number of primate genomes during the last few years [38,55] and our own mRNA/DNA sequencing for several CYP4B1 orthologs, we demonstrated here that several other mutational hits had occurred independently on each other in the *CYP4B1* genes in our direct relatives that all had the same purpose: to completely destroy the enzymatic activity of the CYP4B1 orthologs.

In addition to serine 427, we were able to identify four new distinct genetic mechanisms that all led to diminished enzymatic activity when introduced into reference CYP4B1 orthologs:

1.) The p.V71G substitution found exclusively in Denisovans is located central within the A helix [43] and has a profound negative impact on protein expression and longevity (Figs 5 and S5-S6). Introducing the valine-to-glycine exchange into hCYP4B1 P427 and rCYP4B1 P422 highlighted a more dominant effect caused by p.V71G in the human enzyme. Here, the V71 interacts in a hydrophobic manner with A411, located in the K’ helix lying opposite. Measurements between both residues demonstrated a 4.6 Å distance between both residues, indicating important van der Waals contacts for the formation of secondary structure elements [56]. Thus, the presence of G71 may simply lead to destabilization due to the lack of hydrophobic interactions. In contrast, better stabilization of this region in the rabbit CYP4B1 enzyme seems to occur by the additional H-bond interactions between the polar amino acids S416 and Q75 in the K’ and A helix, respectively. At the corresponding position 75, the glutamic acid in the human enzyme is changed into a histidine (p.Q75H). This results in a further loss of stabilizing interactions around position 71 in the human CYP4B1 protein.
2.) The arginine-to-cysteine exchange at position 106 (p.R106C) found in all apes leads to profound impairment of the enzymatic function when introduced into the rCYP4B1 and a complete inactivation of the *Homo sapiens* CYP4B1 P427 (Fig 6). Vice versa, the p.C106R mutation restored the enzymatic functionality of the ape CYP4B1 orthologs (Fig 7A-C). Interestingly, however, in contrast to p.V71G and p.P427S, the p.R106C substitution does not affect the protein expression levels of CYP4B1 orthologs from great apes (Fig 7D). Position 106 is located in the B-C loop which is part of the substrate access channel, and moreover appears to be superficial in a region of greater flexibility in the protein [45] (Fig 5). Here, the main function of these rather flexible loop-containing regions is to connect the ordered secondary structure elements of α-helices and β-sheets in proteins. Therefore, amino acid substitutions in loop regions are not necessarily associated with protein instability, but they are evolutionary selected regions optimized for greater flexibility of proteins. Often, mutations located in rather flexible regions change functionality, thus leading to a dominant diversification in the catalytical mode of action across evolutional adaptation [57,58]. In CYP4B1, the substrate recognition site (SRS)-1 is located adjacent to the B-C loop, starting just N-terminally of the C helix [43,59], whereby the B-C loop is part of the substrate access channel: molecular dynamics simulations demonstrated that ligands commonly access the substrate binding site via the G and I helices, the B’ helix and the B-C loop. This region is highly variable in sequence between different CYP enzymes and appears to be of high importance for substrate specificity [60,61]. In principle, mutation of amino acids near the SRS site might also interrupt its conserved structure with an adverse effect in substrate binding and/or recognition, thus possibly leading to a shift in substrate recognition or catalytic activity that might be caused by adaptations to the environment and/or chemical exposure.
3.) The arginine-to-histidine alteration at position 244 (p.H244R) in bonobo, orangutan and gibbon is located within the G helix of CYP4B1 and has only slight adverse effects on protein activity. In contrast to position 106, position 244 is located within the ordered structure of the G helix [11,43] and therefore probably has a less dominant effect in evolutionary-based substrate adaption and protein functionality. This hypothesis is in concordance with the finding that the position 244 in CYP4B1 of hominoids shows an arginine in most species, but cysteine in bonobo, orangutan, and gibbon. Further analysis of functional sites, that are in close proximity to 244 and where the p.R244C exchange might have an impact on the catalytic activity, revealed the presence of SRS-3 in other human CYPs [59]. Although SRS-3 is yet not mentioned for CYP4B1 [10], it would be worth to analyze whether systematic substitutions of defined residues within this region might impact substrate binding/recognition, due to a destabilization within functional domains or direct substrate interaction. Our finding that the p.R244H mutations resulted in greater toxicity when PK was used as a substrate may be due to the fact that the CYP4B1 binding channel has several non-polar amino acid residues, which favor more lipophilic substrates.
4.) Due to the exonic deletion identified in the gorilla and the weak splice acceptor of the exon 10 (corresponding to exon 11 in all other hominoids), the deletion not only leads to a truncated enzyme lacking amino acids of the meander region but also causes a frameshift resulting in a premature stop in the last exon. Therefore, this shortened gorilla protein with the p.R106C substitution and no meander region certainly lacks any CYP4B1 enzyme activity.

The fact that multiple genetic hits independently occurred in the same gene in related species is an indication of the importance of inactivating CYP4B1 for hominoid evolution. A similar evolutionary progression is notable for the uricase gene wherein apes, including humans, have lost the ability to metabolize uric acid to the more soluble allantoin [62]. In *Homo sapiens*, the uricase gene possesses two premature stop codons, but simply removing these by mutagenesis does not reactivate the gene. Kratzer *et al.* [62] suggested that more than 20 mutational events over some 20 million years resulted in a progressive loss of enzyme activity. These authors further suggested that the loss of uric acid catabolism, which is a causative feature of gout, might have been offset by the acquisition of a beneficial enzyme activity wherein fructose is rapidly converted into fat. Today, a recombinant baboon-pig chimeric uricase (Rx Pegloticase) is used therapeutically to restore human uricase activity and thereby combat gout [63]. What then might have driven the progressive acquisition of inactivating mutations in CYP4B1?

The complete loss of enzyme function against hallmark substrates of active CYP4B1 orthologs strongly suggests that it was not just a specificity adaptation of CYP4B1 substrates based on evolutionary requirements (behavior, dietary or habitat preferences) or an inhibition of catalytical function due to not yet identified species-specific alterations, but some highly toxic substance(s) in the food or environment that may have enforced complete elimination of any hominoid carrier with an active CYP4B1 enzyme. During evolution, genes coding for proteins participating in metabolism of xenobiotics exhibit less conserved evolutionary processes than genes that encode proteins involved in biosynthesis. Thus, a higher rate of gene duplications, pseudogenization and amino acid changes occurs in the first set of genes [64]. Since CYP4B1 not only has the capacity to hydroxylate fatty acids, but also participates in the metabolism of xenobiotic compounds, it remains striking that there is still a highly conserved amino acid sequence between active and catalytically inactive CYP4B1 enzymes of different primate species. In conclusion, evolution of the *CYP4B1* gene seems to have combined both the characteristics of a gene involved in xenobiotic and endobiotic functions. Thus, elucidating the evolutionary trigger(s) for CYP4B1 inactivation in our ancestors will ultimately improve our understanding of primate evolution.

## Materials and methods

### Cells lines, bacteria, and tissue samples

All already established human cell lines used, the human liver cancer cell lines HepG2 and HuH-7 and the human embryonic kidney cell line HEK293T, were purchased from ATCC (Manassas, VA, USA.), and grown in Dulbeco’s modifies Eagle’s medium supplemented with 5–10% fetal bovine serum, 100 U/ml penicillin and 100 µg/ml streptomycin (Gibco Lifescience, Thermo Fisher Scientific, USA). Both HepG2 and HuH-7 cells, were cultured on 0.1% galantine (Sigma-Aldrich, #G1890) coated plates.

Strain *E. coli* OverExpress C43(DE3) [F^−^ *ompT hsdSB* (*rB*^*-*^ *mB*^*-*^) *gal dcm* (DE3)] (Lucigen Corporation, USA) was used for *cyp4b1* expression.

The German Primate Center, Leibniz Institute for Primate Research provided lung tissue samples from gibbon, orangutan, gorilla, and chimpanzee; all tissue samples were collected from dead animals.

### CYP4B1 sequences

*CYP4B1* cDNA sequences from prosimians, OWMs, bonobo, *Homo sapiens*, and rabbit were obtained from public databases (Ensembl, NCBI): *Carlito syrichta*/Philippine tarsier: XP_008055471.1; *Microcebus murinus*/gray mouse lemur: XP_012631555.1; *Macaca mulatta*/rhesus monkey: ENSMMUT00000047213_3; *Chlorocebus sabaeus*/African green monkey: XM_007978860.2; *Piliocolobus tephrosceles*/Ugandan red colobus: XM_023221208.1; *Rhinopithecus bieti*/Yunnan snub-nosed monkey: XM_017881706.1; *Pan paniscus*/bonobo: ENSPPAT00000044345_1; *Homo sapiens*/modern human (NM_001099772.2); native rabbit (NM_001082103, isoform 1). Sequences from Denisovan and Altai Neanderthal humans were kindly provided by Prof. Dr. Svante Pääbo from the Max Planck Institute for Evolutionary Anthropology, University of Leipzig. Genome data of the Vindija Neanderthal were obtained from the Max Planck Institute for Evolutionary Anthropology (http://ftp.eva.mpg.de/neandertal/Vindija/).

### Chemicals

**Fig 8:**
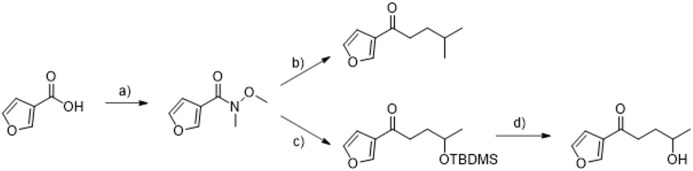
Synthesis of perilla ketone and 4-ipomeanol. a) HNMeOMe, TBTU, HOBt, NEt_3_, DCM, RT; b) Mg, 1-bromo-3-methylbutane, THF, 0 °C to RT; c) Mg, *O*-TBDMS-4-bromo-2-butanol, THF, 0 °C to RT; d) TBAF, THF, RT.

To synthesize PK and 4-IPO we opted for a Weinreb ketone synthesis (Fig 8). The necessary Weinreb amide was synthesized from 3-furancarbonic acid (yield of 87%). The bromides required for the formation of the Grignard reagents were either commercially available or synthesized from 4-hydroxy-2-butanon in a simple 3 step sequence. First, the primary alcohol was transformed into bromide with PBr_3_, followed by reduction of the ketone with NaBH_4_ and finally the protection of the resulting secondary alcohol with *tert*-butyldimethylsilyl chloride. The reaction of *N*-methoxy-*N*-methyl-3-furancarboxamide with 2 equivalents of bromo(3-methylbutyl)magnesium gave PK in 72% yield. The synthesis of 4-IPO, however, required 3 equivalents of the corresponding Grignard reagent to give the TBDMS-protected product in a sufficient yield of 80%. The final deprotection with TBAF gave 4-IPO in 85% yield (**detailed synthesis is explained in** S2 File).

Lauric acid (LA) and undecanoic acid (UA) were purchased from AppliChem (#A5423.0100) and ACROS Organics (#173970050), respectively.

### CYP4B1 identification from lung tissues

#### RT-PCR Analysis.

Total RNA from lung tissues of five gibbons (*Hylobates lar*, *Hylobates* sp., *Nomascus leucogenys*, and *Symphalangus syndactylus*), three gorillas (*Gorilla gorilla*), four orangutans (*Pongo* sp.), and three chimpanzees (*Pan troglodytes*) were isolated using the NucleoSpin RNA Kit (Macherey-Nagel). The samples were roughly chopped on dry ice before being mechanically disrupted and homogenized in lysis buffer. Total RNA isolation was performed according to the manufacture’s protocol. cDNA was synthesized from 1 µg RNA using M-MuLV Reverse Transcriptase (NEB, #M0253L) with random hexamer primers. For amplification of CYP4B1 genes PCR assays were performed CYP4B1 specific primers (S1 Table). For PCR approaches, different primer combinations were applied. PCR results were confirmed by performing agarose gel electrophoreses and sequencing (MicroSynth Seqlab GmbH, Göttingen, Germany).

#### Genomic DNA analysis.

PCR was performed using genomic DNA isolated from gorilla lung tissues. Genomic DNA was isolated using the NucleoSpin Tissue Kit (Macherey-Nagel); isolation was performed according to the manufacture’s protocol. PCR assay mixtures contained 500 ng DNA and 0.5 µM primer specifically binding gorilla CYP4B1 in-between exon 9–12 (S1 Table).

### Plasmid construction

The lentiviral expression vector and the specific lentiviral constructs for the native rabbit CYP4B1 (NM_001082103, isoform 1) and the human CYP4B1 (NM_001099772.2) optimized for human codon usage were previously described [28,40,65]. The CYP4B1 cDNAs of all primate enzymes were synthesized by GeneArt after optimization for human codon usage (Thermo Fisher Scientific, Regensburg, Germany) and then cloned into the lentiviral expression vector. The CYP4B1 proteins were C-terminally fused to EGFP via a GKPVSGEPSAT peptide sequence generated via overlap PCR approaches. Generation of the p.V71G, p.R106C, and p.R244H substitutions were generated via an overlap PCR as described [28], using either rabbit, human, or great ape CYP4B1 wild type cDNAs as templates.

For expression in *E. coli* C43(DE3), the *CYP4B1* cDNAs were amplified via PCR and ligated into plasmid pET22b(+) as described [40]. As previously published by us [40,42], the N-terminal membrane anchors were removed by deletion of amino acids 2–24 (N∆2–24) until a consensus sequence (GFLKL/GFFKL), uniformly located after position 24 in each primate ortholog (S1 File), and alanine was added as a second amino acid. A C-terminal 6x His tag with the amino acid sequence AAALEHHHHHH was added and utilized for IMAC purification and western blot detection.

### Lentiviral production and cell transduction

The human liver cancer cell lines HepG2 and HuH-7 were transduced with Vesicular stomatitis virus G (VSV-G)-pseudotyped lentiviral particles as described [28,66]. 24–48 hours later, the transduced cells were selected with 1.5 µg/ml Puromycin (Gibco).

### Cell proliferation assay

The enzymatic activity of different CYP4B1 enzymes was measured by prodrug turnover, resulting in active cell toxins that induced cell death. To this end, 2x 10^4^ transduced human liver cancer cells were cultured in 50 µl DMEM culture medium in in TC-treated 96-well plates for 2 days. Exposure to defined concentrations of either 4-IPO or PK (2.9, 9, 29, 20, and 290 µM) was performed for the last 24 hrs. In case of PK, 96-well plates were covered with gas permeable membranes (QuickSeal Gas Perm Self Adhensive Sealing Film, iST Scientific) to avoid diffusion of the compound. Following prodrug treatment, CYP4B1 enzymatic activity was analyzed indirectly with the cell viability performing MTS assays (CellTiter 96 Aqueous One Solution Cell Proliferation Assay, Promega) according to the manufacturer’s protocol.

### Protein expression in *E. coli*

Expression of recombinant CYP4B1 orthologs in *E. coli* was performed as described previously [40]. Briefly, cell pellets were resuspended in 50 mM potassium phosphate and the cells disrupted via sonification. For affinity chromatography (IMAC), the soluble protein fraction was incubated with nickel nitrilotriacetic acid-agarose and then poured into a gravity column. After washing, the column-bound CYP4B1 was eluted using elution buffer and concentrated by ultrafiltration (30 kDa molecular weight cut-off). Desalting and removal of detergent and imidazole was performed by a PD MidiTrap G-25 column (GE Healthcare, Germany) that was equilibrated with storage buffer according to the manufacturer’s recommendations. CYP4B1 concentrations were calculated from CO-difference spectra according to Omura and Sato [67]. In addition, full spectra between 200 – 700 nm were recorded (S3 Fig). In cases where a 450 nm absorbance could not be measured, the western blot signal was used to roughly estimate for similar P450 concentrations.

Expression and purification of flavodoxin (YkuN) from *Bacillus subtilis* and flavodoxin reductase (Fdr) from *E. coli* JM109 was carried out as described previously [68]. Bovine cytochrome b_5_ (bCYB5) was added since it has proven to increase the conversion of lauric acid with rabbit CYP4B1 as reported previously by us [15]. Expression of bovine cytochrome bCYB5, cloned into the pET17b plasmid (kindly provided by Prof. Rita Bernhardt [69]) was expressed as described elsewhere [70] and purified via IMAC [71]. Glucose dehydrogenase (GDH) from *Bacillus megaterium* (gdhIV; GenBank D10626) was used for co-factor regeneration. GDH was expressed in *E. coli* BL21(DE3) from pET22b(+) and partially purified by a combination of salt precipitation and heat denaturation of endogenous *E. coli* proteins as described previously [72].

### Reconstitution of CYP4B1 activity against LA and 4-IPO

Catalase from bovine liver was used for scavenging of H_2_O_2_ and was obtained from Sigma-Aldrich (#C1345-10G).

Substrate conversions were carried out in 50 mM KPi, pH 7.5. Reaction mixtures contained 0.25 µM CYP4B1, 2 µM Fdr, 20 µM YkuN, 0.5 µM bCYB5, 1.000 U/ml catalase, 25 U ml^-1^ GDH, 20 mM glucose, 25 µg/ml 1,2-didodecanoyl-*sn*-glycero-3-phosphocholine (DLPC), 200 µM LA or 4-IPO and 200 µM NADPH. Samples were incubated for 90 min at 30 °C under constant movement. Reactions were stopped and acidified by addition of 2 µl 37% HCl, followed by addition of 100 µM C11 or (-)-carvone, used as internal standard for LA or 4-IPO conversion, respectively. LA and its reaction products were extracted two times with 500 µl methyl tert-butyl ether. The organic phases were pooled, dried with water-free MgSO_4_, and evaporated. The residues were dissolved in 30 µl N,O-bis(trimethylsilyl)trifluoroacetamide with 1% trimethylchlorosilane and incubated at 80 °C for 30 min. Reaction mixtures with 4-IPO were stopped and extracted by addition of 100 µl ethyl acetate. Qualitative and quantitative analysis of the samples by GC/MS was performed as described previously for LA [15] and 4-IPO [40].

### Western Blot analysis

We performed immunoblots with samples of whole cell lysates (50 µg of total soluble protein) on 10% SDS-PAA gels as previously described [73]. Following protein transfer onto polyvinylidene difluoride (PVDF) membranes, samples were probed with different antibodies: wild type CYP4B1-EGFP fusion proteins were detected using the mouse anti-GFP primary antibody (1:5000, Clontech); non-tagged human and ape CYP4B1 variants were detected using mouse anti-human CYP4B1 pAb (1:1000, Abcam), and non-tagged rabbit CYP4B1 was detected using goat anti-rabbit CYP4B1 pAb (1:5000, previously published by Parkinson *et al.* [74], 5 mg/ml IgG solution). To detect the primary antibodies via chemiluminescence, HPR-conjugated secondary antibodies were used: goat anti-mouse IgG or rabbit anti-goat IgG (Thermo Fisher Scientific), each diluted 1:2500. The Immobilon Western Reagents (Millipore Corporation) and the ChemoCam Imager (INTAS Science Imaging Instruments GmbH) were used for signal detection. The λ-Tubulin signal of each sample served as a loading control and was measured on the same membranes after stripping and re-probing with mouse anti-λ-Tubulin mAb (1:1000, Merck) and secondary HPR-conjugated antibody (uncropped Western blots are available in S7 Fig).

In addition, we performed immunoblots with samples of the CYP4B1 enzymes expressed in *E. coli* (10 µg protein) on 12.5% bis-tris polyacrylamide gels. Following protein transfer onto nitrocellulose membraned samples were probed with mouse monoclonal 6x His tag antibody (HIS.H8, 1:1,000 diluted, Thermo Fisher Scientific, catalog no. MA1–21315). The secondary horseradish peroxidase-linked goat-anti-mouse polyclonal antibody (1:10,000 diluted, Jackson ImmunoResearch, catalog no. 115-035-003) was utilized to detect the primary antibody.

### Protein stability assay using cycloheximide

To determine protein stability of different CYP4B1 enzymes, HepG2 cells stably expressing these enzymes as CYP4B1-EGFP fusion proteins were used as previously described [28]. One day prior to cycloheximide treatment (final concentration of 50 µg/ml, Sigma-Aldrich), HepG2 cells were seeded in TC-treated 6-well plates with 500,000 cells per well and incubated overnight. Treatment with cycloheximide was stopped after defined time periods (5, 10, 25, and 35 hrs) and the EGFP signal was analyzed by flow cytometry analysis. Half-life measurements of different time points were normalized to untreated controls.

### Microscopy

HuH-7 cells overexpressing CYP4B1 isoforms tagged with EGFP were seeded (8x10^4^ cells per well) on an 8-chamber glass slide (Ibidi, Gräfeling, Germany) coated with 0.1% gelatin. Following attachment, cells were washed one time and fixed by adding a 4% PFA solution for 10 min. Fixed cells were then co-stained for blue nuclei (HOECHST34580, 1:500, Thermo Fisher Scientific) and F-actin (Phalloidin, 1 µg/ml, Sigma-Aldrich) for 40 min at 37 °C. Stained cells were visualized using the ZEISS LSM 880 laser-scanning microscope. Pictures of all samples were performed using a 63x objective with equal adjustments and processed using the Fiji-ImageJ software (version 1.54d).

### Phylogenetic analysis

All CYP4B1 sequences, either obtained from NCBI or identified via RNA sequencing, were further analyzed via CLUSTALW multiple alignment method following a multiple sequence alignment performed with the SnapGene 6.0.7 software (Boston, MA, USA, www.snapgene.com).

To calculate the non-synonymous/synonymous (dN/dS) substitution rate for each codon HyPhy was used. Sequence alignment and a Maximum-likelihood tree [75–77] including all WT CYP4B1 sequences were obtained using MEGA12 software [78]. A standard bootstrapping analysis was conducted using 500 replicated, with other parameters set as default. To test for positive selection within the CYP4B1 evolution the fixed-effects likelihood (FEL) method (analysis version 2.5) that estimates sidewise dN and dS rates was conducted. Overall, 14 sequences and 25 branches were included and P-values < 0.1 were considered to be significant [46]. Detailed description can be found in S4 Data.

### *In-silico* modeling of CYP4B1

The 3D-structure of rabbit CYP4B1 (PDB 5T6Q) was downloaded from PDB (https://www.rcsb.org/). To model the appropriate amino acids V66, R101, and R239, the backbone structure was kept fixed. For mutagenesis of the proteins, N-cap and C-cap were set as open, and rotamers were set as backbone dependent. For p.R239H, a neutral protonation state for HIS was applied. The same software was also applied to analyze intramolecular (polar) interactions related to the appropriate amino acids and their variants.

### Analysis of Splice sites in CYP4B1 enzymes

Genomic CYP4B1 sequences were analyzed for the occurrence of splice sites using the HEXplorer web-interface (https://rna.hhu.de/HEXplorer/) [79]. The strength of splice donor sequences was estimated using the HBond score (HBS), which ranges from 1.8 to 23.8 [80]. The splice acceptor strength was calculated by the MaxEntScan score, which ranges between -37 and 16 [81].

### Statistical analysis

All data presented are shown as mean ± SEM. For statistical analysis, a multiple comparison one-way ANOVA with subsequent Dunnett’s-Post-hoc test was used to determine significant differences between measuring points compared to untreated controls: *p*-values < 0.05 were defined as significant and were marked with an asterix (*) within the graphs. In addition, a non-linear fit model ([Inhibitor] vs. response – Variable slope (four parameters), Prism Version 8.0.2 (GraphPad Software, San Diego, CA, USA)) was used to determine the GI_50_ values of concentration-response data.

## Supporting information

S1 File Amino acid multiple sequence alignment (MSA) of all analyzed CYP4B1 WT sequences. MSA was generated using CLUSTALW, including all analyzed CYP4B1 orthologs. Sequences published in NCBI: *Carlito syrichta*/Philippine tarsier XM008057280.1; *Microcebus murinus/* gray mouse lemur XM_012776101.1; *Rhinopithecus bieti*/Yunnan snub-nosed monkey XM_017881706.1; *Piliocolobus tephrosceles*/Ugandan red colobus XM_023221208.1; *Chlorocebus sabaeus*/African green monkey XM007978860.2 and *Macaca mulatta*/rhesus monkey XM_001108915.4; *Oryctolagus cuniculus*/rabbit NM_001082103, isoform 1, and *Homo sapiens* NM_001099772.2. Prof. Dr. Svante Pääbo kindly provided sequences from Denisovan and Neanderthal. CYP4B1 sequences of great apes were generated via reverse transcription PCR, subsequent sequencing of PCR products, and validation against the information present in public databases.
(PDF)

S2 File Synthesis of 4-IPO and PK. Detailed synthesis of 4-ipomeanol (4-IPO) and perilla ketone (PK).
(PDF)

S1 Fig Systematic analysis of the activity of different CYP4B1 orthologs during primate evolution. CYP4B1 orthologs from different evolutionary lineages (prosimians, Old World monkeys (OWMs), gibbon, great apes, and *Homo sapiens*) were systematically analyzed. **(A-C)** The activity of CYP4B1 isoforms was evaluated performing a MTS assay following a 24 hrs treatment with either 4-ipomeanol, 4-IPO (upper graphs) or Perilla ketone, PK (lower graphs). Experiments were performed in the human liver carcinoma cell lines, HepG2. Graphs are divided based on the evolution of human; left: gibbon and great apes **(A)**, middle: Old World monkeys **(B)**, and right: prosimians **(C)**. For calculation of the half maximal effective concentration reducing cell survival by 50% (GI_50_ values) a nonlinear fit model was applied to each data set and the bottom was set to 0 while the top was set to 100 (S3 Data). For each data set at least three individual replicates were measured and are shown as mean ± SEM. For statistical analysis, a multiple comparison one-way ANOVA with subsequent Dunnett’s-Post-hoc test was used to determine significant differences between measuring points compared to untreated controls: p-values < 0.05 were defined as significant and were marked with an asterix (*). The underlying data for the graphs in this figure can be found in S2 Data.
(TIF)

S2 Fig Artificial engineering of a full-length gorilla CYP4B1. **(A)** cDNA sequence of wild type gorilla CYP4B1 and engineered gorilla CYP4B1 containing human exon 10 and gorilla exon 10 (corresponding to human exon 11) and full-length gorilla exon 11 (corresponding to human exon 12). The amino acid sequence of the 3’ human exon 10 that is identical in all Hominoidea and gorilla exon 10 (corresponding to human exon 11) that differs in two positions from the human exon (marked in orange). **(B)** MTS assay of wild type and engineered gorilla CYP4B1 enzymes. For each data set at least three individual replicates were measured and are shown as mean ± SEM. For statistical analysis, a multiple comparison one-way ANOVA with subsequent Dunnett’s-Post-hoc test was used to determine significant differences between measuring points compared to untreated controls: p-values < 0.05 were defined as significant and were marked with an asterix (*). The underlying data for the graphs in this figure can be found in S2 Data.
(TIF)

S3 Fig Expression analysis of recombinant purified CYP4B1 enzymes. **(A-C)** Absorption spectra of CYP4B1 orthologs including gibbon, great apes and human **(A)**, OWM **(B)**, and prosimians **(C). (D-F)**. CO-difference spectra of CYP4B1 orthologs purified from soluble protein fractions *via* affinity chromatography. In contrast to the analyzed OWMs and prosimians CYP4B1 orthologs of Hominoidea revealed no 450 nm peak, thus only the spectra of chimpanzee and gibbon are shown as representatives. **(G)** SDS-PAGE and **(D)** Western blot analysis of all CYP4B1 orthologs. Membranes were probed with mouse monoclonal 6x His tag antibody (HIS.H8, 1:1,000 diluted, Thermo Fisher Scientific, #MA1–21315). The secondary horseradish peroxidase-linked goat-anti-mouse polyclonal antibody (1:10,000 diluted, Jackson ImmunoResearch, #115-035-003) was utilized to detect the primary antibody *via* a colorimetric peroxidase reaction.
(TIF)

S4 Fig Multiple sequence alignments highlighting the positions relevant for enzymatic activity. **(A)** Analyzed were CYP4B1 orthologs from multiple species described to be active. **(B)** MTS assay of CYP4B1 orthologs described to be active that were tested within our study. **(C)** On the other hand, members of the CYP4 family were analyzed to identify any conservation patterns of the identified key positions relevant for enzyme function. **(D)** Guide tree of human CYP4 enzymes created with ClustalW. The underlying data for the graphs in this figure can be found in S2 Data and the respective GI_50_ values for each CYP4B1 ortholog can be found in S3 Data.
(TIF)

S5 Fig Functional analysis of mutated CYP4B1 enzymes in HepG2 cells. **(A)** Activities of *Homo sapiens* wild type (S427; WT) and ‘re-activated’ variant p.S427P (P427), as well as Denisovan wild type (S427) and Denisovan mutant p.S427P. (**B**) p.V66G was introduced and analyzed in rabbit wild type (P422; WT) and mutant p.P422S (S422). **(C)** In addition, p.R106C and p.R244H were systematically analyzed in *Homo sapiens*, **(D)** rabbit (corresponding to p.R101C and pR239H), and exemplified great apes (bonobo **(E)** and orangutan **(F)**, and gibbon **(G)**). Bioactivation of 4-IPO or PK was measured via MTS assay following a 24 hrs treatment; based on a nonlinear fit the respective GI_50_ values were calculated (S3 Data). All experiments were performed with a minimum of three individual replicates. For each data set at least three individual replicates were measured and are shown as mean ± SEM. For statistical analysis, a multiple comparison one-way ANOVA with subsequent Dunnett’s-Post-hoc test was used to determine significant differences between measuring points compared to untreated controls: p-values < 0.05 were defined as significant and were marked with an asterix (*). The underlying data for the graphs in this figure can be found in S2 Data.
(TIF)

S6 Fig FACS measurement to analyze any effects of the p.V71G on protein stability. **(A)** FACS measurements to analyze any effects of the p.V71G (corresponding to p.V66G in rabbit) on protein stability. **(B)** Calculated half-life of the respective proteins. The underlying data for the graphs in this figure can be found in S2 Data.
(TIF)

S7 Fig Raw western blot images. **(A)** Western Blots of CYP4B1-EGFP fusion proteins. For detection, an anti-EGFP antibody was used. **(B)** Effect of the amino acid exchanges p.P427S, p.V71G, p.R106C and p.R244H on the expression of *Homo sapiens* and **(C)** rabbit (wild type and p.P422S variant) CYP4B1. Human CYP4B1 enzymes were detected with an anti-human CYP4B1 polyclonal antibody. Rabbit CYP4B1 enzymes were detected with an anti-rabbit CYP4B1 polyclonal antibody. **(D)** Effect of the amino acid exchanges p.C106R and p.H244R on expression levels of gibbon, orangutan, and bonobo CYP4B1 proteins in HuH-7 cells. The hominoid proteins were detected with an anti-human CYP4B1 antibody. γ-tubulin served as a loading control for all performed western blots.
(TIF)

S1 Data The underlying date for the graphs (Figs 1–7). (XLSX)

S2 Data The underlying data for the graphs (S1-S6 Figs). (XLSX)

S3 Data GI_50_ values and 95% Confidence intervals. Based on a non-linear curve fit model the GI_50_ values and the respective 95% CI intervals for each CYP4B1 ortholog analyzed were calculated; thereby, the bottom was set to zero while the top was set to 100; n.r. = not reached.
(XLSX)

S4 Data Analysis of positive selection using FEL (fixed-effects likelihood) method. Site-by-site results compromising dN/dS results for each codon with P-values < 0.1 were considered significant. Detailed description of the analysis method and the maximum-likelihood tree building the basis for FEL analysis.
(XLSX)

S1 Table Oligonucleotides designed for sequencing CYP4B1 orthologs from apes, introducing amino acid changes, and establishing CYP4B1-EGFP fusion proteins. (XLSX)

S1 Text Detailed description of the sequence analysis of gorilla CYP4B1. (PDF)
