# Supplementary material for: Functional analysis of CYP4B1 enzymes from apes and humans uncovers evolutionary hot spots for adaptations of the catalytical function
Source: PLoS Genet. 2025 Jun 27;21(6):e1011750. doi: 10.1371/journal.pgen.1011750 (PMC12233900; doi:10.1371/journal.pgen.1011750)
Supplement: S1 File — MSA was generated using CLUSTALW, including all analyzed CYP4B1 orthologs. Sequences published in NCBI: Carlito syrichta/Philippine tarsier XM008057280.1; Microcebus murinus/ gray mouse lemur XM_012776101.1; Rhinopithecus bieti/Yunnan snub-nosed monkey XM_017881706.1; Piliocolobus tephrosceles/Ugandan red colobus XM_023221208.1; Chlorocebus sabaeus/African green monkey XM007978860.2 and Macaca mulatta/rhesus monkey XM_001108915.4; Oryctolagus cuniculus/rabbit NM_001082103, isoform 1, and Homo sapiens NM_001099772.2. Prof. Dr. Svante Pääbo kindly provided sequences from Denisovan and Neanderthal. CYP4B1 sequences of great apes were generated via reverse transcription PCR, subsequent sequencing of PCR products, and validation against the information present in public databases. (PDF) [file pgen.1011750.s001.pdf]

Supplements

```
Homo sapiens                  MVPSFLSLSFSSLGLWASGLILVLGFLKLIHLLLRRQTLAKAMDKFPGPPTHWLFGHALE      60
Neanderthal                   MVPSFLSLSFSSLGLWASGLILVLGFLKLIHLLLRRQTLAKAMDKFPGPPTHWLFGHALE      60
Denisovan                     MVPSFLSLSFSSLGLWASGLILVLGFLKLIHLLLRRQTLAKAMDKFPGPPTHWLFGHALE      60
Chimpanzee                    MVPSFLSLSFSSLGLRASGLILVLGFLKLIRLLLRRQMLAKAMDNFPGPPTHWLFGHALE      60
Bonobo                        MVPSFLSLSFSSLGLWASGLILVLGFLKLIRLLLRRQMLAKAMDNFPGPPTHWLFGHALE      60
Orangutan                     MVPKFLSLSFSSLGLWASGLILVLGFLKLIRLLLRRQMLAKAMDNFPGPPTHWLFGHALE      60
Gorilla                       MVPSFLSLSFSSLGLWASGLILVLGFLKLIRLLLRRQMLAKAMDNFPGPPTHWLFGHALE      60
Gibbon                        MVPSFLSLSFSSLGLWASGLILVLGFLKLIRLLLRRQMLAKAMDNFPGPPTHWLFGHALE      60
Rhesus Monkey                 MVPSFLSLRLSCLGLWASGLILVLGFLKLIRLLLRRQRLAKAMGNFPGPPTHWLFGHALE      60
African green Monkey          MVPSFLSLRLSCLGLWASGLILVLGFLKLIRLLLRRQRLAKAMGNFPGPPTHWLFGHALE      60
Yunnan Snub-nosed Monkey      MVPSFLSLRLSCLGLWASGLILVLGFLKLIGLLLRRQRLAKAMGNFPGPPTHWLFGHALE      60
Red colobus                   MVPSFFSLRLSCLGLWASGLILVLGFLKLIRLLLRRQRLAKAMGNFPGPPTHWLFGHALE      60
Tarsier                       MVPGFLSLSLSRLGLWASGLVLVLGFFKLIHLLLRKQKLARAMDSFPGPPTHWLFGHTLE      60
Mouse lemur                   MVPGFLSPSLSRLGLWASGLILILGFLKLISLLLRRQKLARAMENFPGPPTHWLFGHALE      60
Rabbit                        -----MLGFLSRLGLWASGLILILGFLKLLRLLLRRQRLARAMDSFPGPPTHWLFGHALE      55

Homo sapiens                  IQETGSLDKVVSWAHQFPYAHPLWFGQFIGFLNIYEPDYAKAVYSRGDPKAPDVYDFFLQ      120
Neanderthal                   IQETGSLDKVVSWAHQFPYAHPLWFGQFIGFLNIYEPDYAKAVYSRGDPKAPDVYDFFLQ      120
Denisovan                     IQETGSLDKGGSWAHQFPYAHPLWFGQFIGFLNIYEPDYAKAVYSRGDPKAPDVYDFFLQ      120
Chimpanzee                    IQETGSLDKVVSWAHQFPYAHPLWFGQFIGFLNIYEPDYAQAVYSCGDPKAPDVYDFFLQ      120
Bonobo                        IQETGSLDKVVSWAHQFPYAHPLWFGQFIGFLNIYEPDYAKAVYSCGDPKAPDVYDFFLQ      120
Orangutan                     IQETGSLDKVVSWAHQFPYAHPLWFGQFIGFLNIYEPDYAKAVYSCGDPKAPDVYDFFLQ      120
Gorilla                       IQETGSLDKVVSWAHQFPYAHPLWFGQFIGFLNIYEPDYAKAVYSCGDPKAPDVYDFFLQ      120
Gibbon                        IQETGSLDKVVSWAHQFPYAHPLWFGQFIGFLNIYEPDYAKAVYSCGDPKAPDVYDFFLQ      120
Rhesus Monkey                 IQQTGSLDKVVSWAHQFPYAHPLWFGQFIGFLNIYEPDYAKAVYSRGDPKAPDVYDFFLQ      120
Yunnan African green Monkey   IQQTGGLDKVVSWAHQFPYAHPLWFGQFIGFLNIYEPDYAKAVYSRGDPKAPDVYDFFLQ      120
Snub-nosed Monkey             IQQTGSLDKVVSWAHQFPYAHQLWFGQFIGFLNIYEPDYAKAVYSRGDPKAPDVYDFLLQ      120
Red colobus                   ILQTGSLDKVVSWAHQFPYAHQLWFGQFIGFLNIYEPDYAKAVYSRGDPKAPDVYDFLLQ      120
Tarsier                       IQQTGSLDKVVSWAHQYPCAHPLWFGPFIGFLNIYEPDFAKAVYSRGDPKASDVYDFFLQ      120
Mouse lemur                   IQQTGSLDKVVSWAHQFPYAHPLWFGQFIGFLNIYEPDYAKAVYSRGDPKAADVYDFFLQ      120
Rabbit                        IQKTGSLDKVVTWTQQFPYAHPLWVGQFIGFLNIYEPDYAKAVYSRGDPKAPDVYDFFLQ      115

Homo sapiens                  WIGRGLLVLEGPKWLQHRKLLTPGFHYDVLKPYVAVFTESTRIMLDKWEEKAREGKSFDI      180
Neanderthal                   WIGRGLLVLEGPKWLQHRKLLTPGFHYDVLKPYVAVFTESTRIMLDKWEEKAREGKSFDI      180
Denisovan                     WIGRGLLVLEGPKWLQHRKLLTPGFHYDVLKPYVAVFTESTRIMLDKWEEKAREGKSFDI      180
Chimpanzee                    WIGRGLLVLEGPKWLQHRKLLTPGFHYDVLKPYVAVFTESTRIMLDKWEEKAREGKSFDI      180
Bonobo                        WIGRGLLVLEGPKWLQHRKLLTPGFHYDVLKPYVAVFTESTRIMLDKWEEKAREGKSFDI      180
Orangutan                     WIGRGLLVLEGPKWLQHRKLLTPGFHYDVLKPYVAVFTESTRIMLDKWEEKAREGKSFDI      180
Gorilla                       WIGRGLLVLEGPKWLQHRKLLTPGFHYDVLKPYVAVFTESTRIMLDKWEEKAREGKSFDI      180
Gibbon                        WIGRGLLVLEGPKWLQHRKLLTPGFHYDVLKPYVAVFTESTRIMLDKWEEKAREGKSFDI      180
Rhesus Monkey                 WIGRGLLVLEGPKWFQHRKLLTPGFHYDVLKPYVALFAESTRVMLDKWEEKAQEGKSFDI      180
Yunnan African green Monkey   WIGRGLLVLEGPKWFQHRKLLTPGFHYDVLKPYVALFAESARVMLDKWEEKAQEGKSFDI      180
Snub-nosed Monkey             WIGRGLLVLEGPKWFQHRKLLTAGFHYDVLKPYVALFAESTRVMLDKWEEKAQEGKSFDI      180
Red colobus                   WTGRGLLVLEGPKWFQHRKLLTAGFHYDVLKPYVALFAESTRVMLDKWEEKAQEGKSFDI      180
Tarsier                       WIGKGLLVLEGPKWFQHRKLLTPGFHYDVLKPYVSVFAESTRIMLDKWEEKAREDKCFDI      180
Mouse lemur                   WIGKGLLVLEGPKWFQHRKLLTPGFHYDVLKPYVAVFAESTSDMLDKWEEKAREDKSFDI      180
Rabbit                        WIGKGLLVLDGPKWFQHRKLLTPGFHYDVLKPYVAIFADSTRIMLEKWEKKACEGKSFDI      175

Homo sapiens                  FCDVGHMALNTLMKCTFGRGDTGLGH-RDSSYYLAVSDLTLLMQQRLVSFQYHNDFIYWL      239
Neanderthal                   FCDVGHMALNTLMKCTFGRGDTGLGH-RDSSYYLAVSDLTLLMQQRLVSFQYHNDFIYWL      239
Denisovan                     FCDVGHMALNTLMKCTFGRGDTGLGH-RDSSYYLAVSDLTLLMQQRLVSFQYHNDFIYWL      239
Chimpanzee                    FCDVGHMALNTLMKCTFGRGDTGLGHSRDSSYYLAVSDLTLLMQQRLVSFQYHNDFIYWL      240
Bonobo                        FCDVGHMALNTLMKCTFGRGDTGLGH-RDSSYYLAVSDLTLLMQQRLVSFQYHNDFIYWL      239
Orangutan                     FCDVGHMALNTLMKCTFGRGDTGLGH-RDSSYYLAVSDLTLLMQQRLVSFQYHNDFIYWL      239
Gorilla                       FCDVGHMALNTLMKCTFGRGDTGLGH-RDSSYYLAVSDLTLLMQQRLVSFQYHNDFIYWL      239
Gibbon                        FCDVGHMALNTLMKCTFGRGDTGLGH-RDSSYYLAVSDLTLLMQQRLVSFQYHNDFIYWL      239
Rhesus Monkey                 FCDVGHMALNTLMKCTFGRGDTGLGH-RDSSYYLAVSDLTLLMQQRLVSFHYHNDFIYWL      239
Yunnan African green Monkey   FCDVGHMALNTLMKCTFGRGDTGLGH-RDSSYYLAVSDLTLLMQQRLVSFHYHNDFIYWL      239
Snub-nosed Monkey             FCDVGHMTLDTLMKCIFGRGDTGLGH-RDSSYYLAVSDLTLLAQQRLASFHYHNDFIYWL      239
Red colobus                   FCDVGHMALDTLMKCIFGRGDTGLGH-RDSSYYLAVSDLTLLTQQRLVSFYYHNDFIYWL      239
Tarsier                       FCDVGQMALDTLMKCTFGKGDSGLGH-RDSSYYLAVSNLTLLMQQRLESFQYHNDFIYWL      239
Mouse lemur                   FSDVGHMALDSLMKCTFGKGDSGLGQ-RDNSYYLAVSDLTMLMQQRMESFQYHNDFIYWL      239
Rabbit                        FSDVGHMALDTLMKCTFGKGDSGLNH-RDSSYYVAVSELTLLMQQRIDSFQYHNDFIYWL      234

Homo sapiens                  TPHGRRFLRACQVAHDHTDQVIRERKAALQDEKVRKKIQNRRHLDFLDILLGARDEDDIK      299
Neanderthal                   TPHGRRFLRACQVAHDHTDQVIRERKAALQDEKVRKKIQNRRHLDFLDILLGARDEDDIK      299
Denisovan                     TPHGRRFLRACQVAHDHTDQVIRERKAALQDEKVRKKIQNRRHLDFLDILLGARDEDDIK      299
Chimpanzee                    TPHGRRFLRACQVAHDHTDQVIRERKAALQDEKVQKKIQNRRHLDFLDILLGAWDEDDIK      300
Bonobo                        TPHGHRFLRACQVAHDHTDQVIRERKAALQDEKVRKKIQNRRHLDFLDILLGARDEDDIK      299
Orangutan                     TPHGHRFLRACQVAHDHTDQVIRERKAALQDEKVRKKIQNRRHLDFLDILLGARDEDDIK      299
Gorilla                       TPHGRRFLRACQVAHDHTDQVIRERKAALQDEKVRKKIQNRRHLDFLDILLGARDEDDIK      299
```

Gibbon	TPHG**H**RFLRACQVAHDHTDQVIRERKAALQDEKVRKKIQNRRHLDFLDILLGARDEDDIK	299
Rhesus Monkey	TPHG**R**RFLRACQVAHDHTDQVIRERKAALQDEKVRKKIQNRRHLDFLDILLGARDEDDSK	299
Yunnan African green Monkey	TPHG**R**RFLRACQVAHDHTDQVIKERKAALQDEKVRKKIQNRRHLDFLDILLGARDEDDSK	299
Snub-nosed Monkey	TPHG**R**RFLRACQVAHDHTDQVIRERKAALQDEKVRKKTQNRRHLDFLDILLGARDEDDSK	299
Red colobus	TPHG**R**RFLRACQVAHDHTDQVIRERKAALQDEKVRKKTQNRRHLDFLDILLGVQDEDDSK	299
Tarsier	TPHG**R**RFLRACQVAHDHTDQVIRERKAALQDEKEREKIQNRRHLDFLDILLGARDEDGIK	299
Mouse lemur	TPHG**R**RFLRACQVAHDHTDQVIRERKAALQDEKEQRKIQNRRHLDFLDILLGARDGDGIK	299
Rabbit	TPHG**R**RFLRACRAAHDHTDRVIRQRKAALQDEKEREKIQNRRHLDFLDILLDVRGESGVQ	294

Homo sapiens	LSDADLRAEVDTFMFEGHDTTTSGISWFLYCMALYPEHQHRCREEVREILGDQDFFQWDD	359
Neanderthal	LSDADLRAEVDTFMFEGHDTTTSGISWFLYCMALYPEHQHRCREEVREILGDQDFFQWDD	359
Denisovan	LSDADLRAEVDTFMFEGHDTTTSGISWFLYCMALYPEHQHRCREEVREILGDQDFFQWDD	359
Chimpanzee	LSDADLRAEVDTFMFEGHDTTTSGISWFLYCMALYPEHQHRCREEVREILGDQDSFQWDD	360
Bonobo	LSDADLRAEVDTFMFEGHDTTTSGISWFLYCMALYPEHQHRCREEVREILGDQDSFQWDD	359
Orangutan	LSDADLRAEVDTFMFEGHDTTTSGISWFLYCMALYPEHQHRCREEVREILGDQDSFQWDD	359
Gorilla	LSDADLRAEVDTFMFEGHDTTTSGISWFLYCMALYPEHQHCCREEVREILGDQDSFQWDD	359
Gibbon	LSDADLRAEVDTFMFEGHDTTTSGISWFLYCMALYPEHQHRCREEVREILGDQDSFQWDD	359
Rhesus Monkey	LSDADLRAEVDTFMFEGHDTTTSGISWFLYCMALYPEHQHRCREEVREILGDQDSFQWDD	359
Yunnan African green Monkey	LSDADLRAEVDTFMFEGHDTTTSGISWFLYCMALYPEHQHRCREEVREILGDQDSFQWDD	359
Snub-nosed Monkey	LSDADLRAEVDTFMFEGHDTTTSGISWFLYCMALYPEHQHRCREEVREILGDQDSFQWDD	359
Red colobus	LSDADLRAEVDTFMFEGHDTTTSGISWFLYCMALYPEHQHRCREEVREILGDQDSFQWDD	359
Tarsier	LSDMDLRSEVDTFMFEGHDTTTSGISWFLYCMALYPEHQQRCREEALEILGDRDAFQWDD	359
Mouse lemur	LSDADLRAEVDTFMFEGHDTTTSGISWFLYCMARYPEHQQRCRDEAREILGDRDSFQWDD	359
Rabbit	LSDTDLRAEVDTFMFEGHDTTTSGISWFLYCMALYPEHQQRCREEVREILGDQDSFQWED	354

Homo sapiens	LGKMTYLTMCIKESFRLYPPVPQVYRQLSKPVTFVDGRSLPAGSLISMHIYALHRNSAVW	419
Neanderthal	LGKMTYLTMCIKESFRLYPPVPQVYRQLSKPVTFVDGRSLPAGSLISMHIYALHRNSAVW	419
Denisovan	LGKMTYLTMCIKESFRLYPPVPQVYRQLSKPVTFVDGRSLPAGSLISMHIYALHRNSAVW	419
Chimpanzee	LGKMTYLTMCIKESFRLYPPVPQVYRQLSKPVTFVDGRSLPAGSLISMHIYALHRNSAVW	420
Bonobo	LGKMTYLTMCIKESFRLYPPVPQVYRQLSKPVTFVDGRSLPAGSLISMHIYALHRNSAVW	419
Orangutan	LGKMTYLTMCIKESFRLYPPVPQVYRQLSKPVTFVDGRSLPAGSLISMHIYALHRNSAVW	419
Gorilla	LGKMTYLTMCIKESFRLYPPVPQVYRQLSKPVTFVDGRSLPAGTALGSSLP---------	410
Gibbon	LGKMTYLTMCIKESFRLYPPVPQVYRQLSKPVTFVDGRSLPAGSLISMHIYALHRNSAVW	419
Rhesus Monkey	LGKMTYLTMCIKESFRLYPPVPQVYRQLSKPVTFVDGRSLPAGSLISMHIYALHRNSAVW	419
Yunnan African green Monkey	LGKMTYLTMCIKESFRLYPPVPQVYRQLSKPVTFVDGRSLPAGSLISMHIYALHRNSAVW	419
Snub-nosed Monkey	LGKMTYLTMCIKESFRLYPPVPQVYRQLSKPVTFVDGRSLPAGSLISMHIYALHRNSAVW	419
Red colobus	LGKMTYLTMCIKESFRLYPPVPQVYRQLSKPVTFVDGRSLPAGSLISMHIYALHRNSAVW	419
Tarsier	LGKMTYLTMCIKESFRLYPPVPQVYRQLSKPVTFVDGRSLPAGSLISLHIYALHRNSAVW	419
Mouse lemur	LSKMTYLTMCIKESMRLYPPVPQVYRQLSKPVTFVDGRSLPAGSLVSLHIYALHRNSAAW	419
Rabbit	LAKMTYLTMCMKECFRLYPPVPQVYRQLSKPVSFVDGRSLPAGSLISLHIYALHRNSDVW	414

Homo sapiens	PDPEVFD**S**LRFSTENASKRHPFAFMPFSAGPRNCIGQQFAMSEMKVVTAMCLLRFEFSLD	479
Neanderthal	PDPEVFD**S**LRFSTENASKRHPFAFMPFSAGPRNCIGQQFAMSEMKVVTAMCLLRFEFSLD	479
Denisovan	PDPEVFD**S**LRFSTENASKRHPFAFMPFSAGPRNCIGQQFAMSEMKVVTAMCLLRFEFSLD	479
Chimpanzee	PDPEVFD**P**LRFSTENASKRHPFAFMPFSAGPRNCIGQQFAMSEMKVVTAMCLLRFEFSLD	480
Bonobo	PDPEVFD**P**LRFSTENASKRHPFAFMPFSAGPRNCIGQQFAMSEMKVVTAMCLLHFEFSLD	479
Orangutan	PDPEVFD**P**LRFSTENASKRHPFAFMPFSAGPRNCIGQQFAMSEMKVVTAMCLLRFEFSLD	479
Gorilla	--	410
Gibbon	PDPEVFD**P**LRFSTENASKRHPFAFMPFSAGPRNCIGQQFAMSEMKVVTAMCLLRFEFSLD	479
Rhesus Monkey	PDPEVFD**P**LRFSTENASKRHPFAFMPFSAGPRNCIGQQFAMSEMKVVTAMCLLHFEFSLD	479
Yunnan African green Monkey	PDPEVFD**P**LRFSTENASKRHPFAFMPFSAGPRNCIGQQFAMSEMKVVTAMCLLRFEFSLD	479
Snub-nosed Monkey	PDPEVFD**P**LRFSIENASKRHPFAFMPFSAGPRNCIGQQFAMSEMKVVTAMCLLHFEFSLD	479
Red colobus	PDPEVFD**P**LRFSTENASKRHPFAFMPFSAGPRNCIGQQFAMTEMKVVTAMCLLHFEFSLD	479
Tarsier	PDPEVFD**P**LRFSPENVSRRHPFAFMPFSAGPRNCIGQQFAMNEMKVVTALCLLRFEFSLD	479
Mouse lemur	PDPEVFD**P**LRFSPENASGRHPFAFMPFSAGPRNCIGQQFAMNEMKVVTALCLLRFEFCLD	479
Rabbit	PDPEVFD**P**LRFSPENSSGRHPYAFIPFSAGPRNCIGQQFAMNEMKVVTALCLLRFEFSVD	474

Homo sapiens	PSRLPIKMPQLVLRSKNGFHLHLKPLGPGSGK	511
Neanderthal	PSRLPIKMPQLVLRSKNGFHLHLKPLGPGSGK	511
Denisovan	PSRLPIKMPQLVLRSKNGFHLHLKPLGPGSGK	511
Chimpanzee	PSRLPIKMPQLVLRSKNGFHLHLKPLGPGSGK	512
Bonobo	PSRLPIKMPQLVLRSKNGFHLHLKPLGPGSGN	511
Orangutan	PSRLPIKMPQLVLRSKNGFHLHLKPLGPGSGK	511
Gorilla	--------------------------------	410
Gibbon	PSRLPIKMPQLVLRSKNGFHLHLKPLGPGSGK	511
Rhesus Monkey	PSRLPIKMLQLVLRSKNGIHLHLKPLGPGSGK	511
Yunnan African green Monkey	PSRLPIKMLQLVLRSKNGIHLHLKPLGPGSGK	511
Snub-nosed Monkey	PSRLPIKMLQLVLRSKNGIHLHLKPLGPGSGK	511
Red colobus	PSRLPIKMLQLVLRSKNGIHLHLKPLGPGSGK	511
Tarsier	PSRLPIKIPQLILRSKNGIHLHLKPLGPGSGK	511
Mouse lemur	PSRLPIKALRLILRSENGIHLRLKSLRPGSGK	511
Rabbit	PLRLPIKLPQLVLRSKNGIHLYLKPLGPK---	503
