# Supplementary material for: Functional analysis of CYP4B1 enzymes from apes and humans uncovers evolutionary hot spots for adaptations of the catalytical function
Source: PLoS Genet. 2025 Jun 27;21(6):e1011750. doi: 10.1371/journal.pgen.1011750 (PMC12233900; doi:10.1371/journal.pgen.1011750)
Supplement: S2 File — Detailed synthesis of 4-ipomeanol (4-IPO) and perilla ketone (PK). (PDF) [file pgen.1011750.s002.pdf]

Synthesis of PK and 4-IPO

N-methoxy-*N*-methyl-3-furancarboxamide

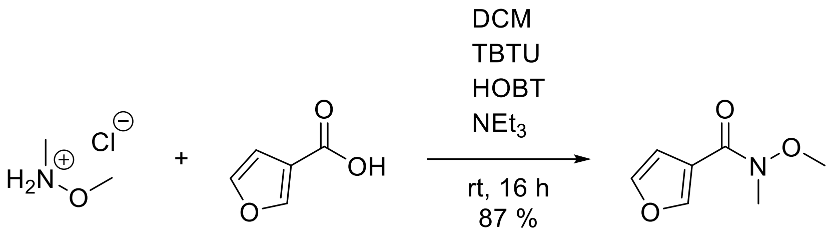

1000 mg (8.92 mmol, 1 eq) furan-3-caboxylic acid where dissolved in 40 ml of dry DCM. 2.047 mg (13.37 mmol, 1.5 eq) HOBT and 4.293 mg (13.37 mmol, 1.5 eq) TBTU followed by 2.247 mg (22.25 mmol, 2.5 eq) NEt_3 where added and the solution was stirred for 30 min at room temperature. 951 mg (9.80 mmol, 1.1 eq) of *N,O*-dimethylhydroxylamin hydrochloride where added and the mixture was stirred for 16 h at room temperature. 50 ml saturated $NaHCO_{3,\ aq}$ where added and the phases separated. The organic phase was washed with 50 ml of $KHSO_{4,\ aq}$ (150 g/L), followed by 50 ml of saturated $NaHCO_{3,\ aq}$ and 50 ml of brine. The organic phase was dried over $NaSO_4$ and the solvent was removed in vacuo. The crude product was purified by flash column chromatography (Cyclohexane:Ethylacetate; 5:1). The product was obtained as a colourless oil in 90 % yield (1.256 g, 8.03 mmol). ^{1}H NMR (400 MHz, $CDCl_3$): δ [ppm] = (dd, J = 1.5, 0.7 Hz, 1H), 7.40 (t, J = 1.7 Hz, 1H), 6.85 (dd, J = 1.9, 0.7 Hz, 1H), 3.69 (s, 3H), 3.32 (s, 3H). ^{13}C-NMR (100.6 MHz, $CDCl_3$): δ [ppm] = 163.24, 146.57, 142.73, 119.83, 111.43, 61.19, 32.87. (ESI): *m/z* = 178.0474 $[M+Na]^+$ (calculated for $C_7H_9NO_2 + Na^+$: 178.0476)

Perilla ketone

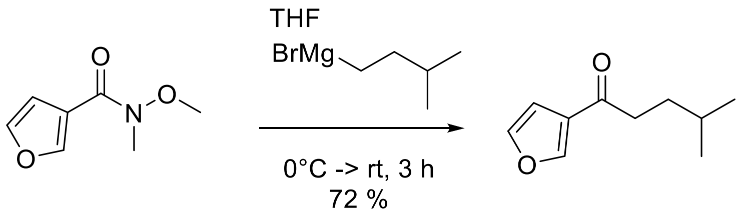

In a 100 ml three necked flask with attached condenser and dropping funnel 196 mg (8.05 mmol, 2.5 eq) of magnesium where activated with iodine and overlaid with 1 ml of dry THF. 973 mg (6.44 mmol, 2 eq) of 1-bromo-3-methylbutane in 10 ml of dry THF where added dropwise, maintaining a mild simmer. The solution was stirred at room temperature for 30 min. At 0 °C 500 mg of *N*-methoxy-*N*-methyl-3-furancarboxamide in 10 ml of dry THF where added dropwise and the mixture was stirred for 3 h at room temperature. The reaction was quenched by the addition of 50 ml saturated $NH_4Cl_{\ aq}$. The phases where separated and then aqueous phase was extracted with 3x30 ml ethyl acetate. The combined organic phases where dried over Na_2SO_4 and

the solvent removed in vacuo. The crude product was purified by flash column chromatography (cyclohexane: ethyl acetate 10:1 -> 5:1). The product was obtained as a colourless oil in 72 % yield (386 mg, 2.32 mmol). ^{1}H NMR (400 MHz, $CDCl_3$): δ [ppm] = 8.03 – 7.99 (m, 1H), 7.42 (t, J = 1.6 Hz, 1H), 6.76 (dd, J = 1.9, 0.8 Hz, 1H), 2.77 – 2.67 (m, 2H), 1.64 – 1.54 (m, 3H), 0.92 (d, J = 6.2 Hz, 6H).^{13}C-NMR (100.6 MHz, $CDCl_3$): δ [ppm] = 195.63, 147.10, 144.26, 127.87, 108.82, 38.66, 33.36, 27.93, 22.50. (ESI): *m/z* = 189.0888 $[M+Na]^+$ (calculated for $C_{10}H_{14}O_2 + Na^+$: 189.0886

4-Bromoethylmethylketone

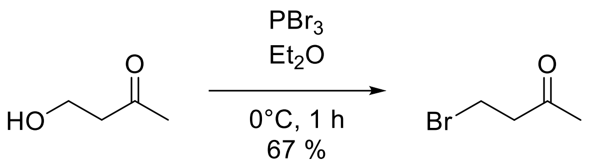

5.00 g (56.7 mmol, 1 eq) 4-hydroxy-2-butanon where dissolved in 50 ml dry Et_2O and 18.43 g (68.0 mmol, 1.2 eq) PBr_3 where added dropwise at 0 °C. The solution was stirred for 1 h at 0 °C and carefully quenched with saturated $NHCO_{3,\,aq}$. The phases where separated and the aqueous phase was extracted with 3x30 ml Et_2O. The combined organic phases where washed with brine, dried over Na_2SO_4 and the solvent was removed in vacuo. The product was obtained as a dark brown liquid in 67 % (5.69 g, 37.7 mmol) yield. ^{1}H NMR (400 MHz, $CDCl_3$): δ [ppm] = 3.54 (t, J = 6.7 Hz, 2H), 3.03 (t, J = 6.7 Hz, 2H), 2.19 (s, 3H). ^{13}C-NMR (100.6 MHz, $CDCl_3$): δ [ppm] = 205.45, 46.15, 30.33, 25.26.

4-Bromo-2-butanol

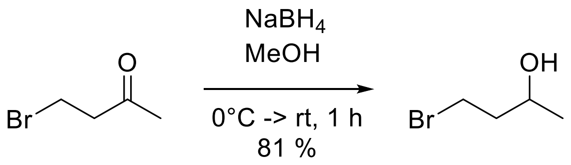

2.00 g (13.25 mmol, 1 eq) 4-bromoethylmethylketone where dissolved in 20 ml dry MeOH and 1.00 g (26.50 mmol, 2 eq) $NaBH_4$ where added carefully at 0 °C. The solution was stirred for 2 h at room temperature and afterwards quenched with H_2O. 20 ml Et_2O where added and the phases separated. The aqueous phase was extracted with 2x20 ml Et_2O and the combined organic phases where washed with brine and dried over Na_2SO_4. The solvent was removed in vacuo and the product obtained as a dark brown liquid in 81 % (1.65 g, 10.78 mmol) yield. ^{1}H NMR (400 MHz, $CDCl_3$): δ [ppm] = 4.09 – 3.96 (m, 1H), 3.60 – 3.44 (m, 2H), 2.15 (s, 1H), 2.03 – 1.92 (m, 2H), 1.24 (d, J = 6.2 Hz, 3H). ^{13}C-NMR (100.6 MHz, $CDCl_3$): δ [ppm] = 66.26, 41.60, 30.47, 23.59.

O-TBDMS-4-Bromo-2-butanol

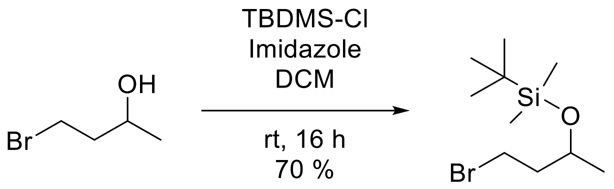

1.60 g (10.46 mmol, 1 eq) 4-bromo-2-butanol where dissolved in 30 ml dry DCM and 1.89 g (12.55 mmol, 1.2 eq) TBDMS-Cl, followed by 1.78 g (26.15 mmol, 2.5 eq) imidazole where added. The solution was stirred for 16 h at room temperature. 50 ml of H_2O where added, the phases separated and the aqueous phase was extracted with 3x30 ml DCM. The combined organic phases where dried over Na_2SO_4 and the solvent removed in vacuo. The crude product was purified by flash column chromatography (Pentan:TBME; 100:1). The product was obtained as colourless oil in 82 % (2.301 g, 8.61 mmol) yield. ^{1}H NMR (400 MHz, $CDCl_3$): δ [ppm] = 4.04 – 3.94 (m, 1H), 3.50 – 3.42 (m, 2H), 2.02 – 1.84 (m, 2H), 1.16 (d, J = 6.1 Hz, 3H), 0.89 (s, 9H), 0.08 (d, J = 4.4 Hz, 6H). ^{13}C-NMR (100.6 MHz, $CDCl_3$): δ [ppm] = 66.49, 42.60, 30.95, 25.99, 23.85, 18.17, -4.14, -4.64.

O-TBDMS-4-Ipomeanol

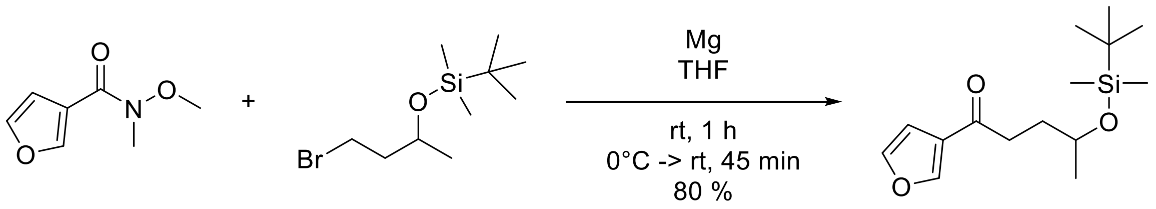

73 mg (3.0 mmol, 3 eq) magnesium where covered with 0.3 ml of dry THF and 802 mg (3.00 mmol, 3 eq) *O*-TBDMS-4-bromo-2-butanol dissolved in 1 ml of dry THF where added dropwise. The reaction was stirred for 1 h maintaining a mild simmer, until all the magnesium had vanished. Further 10 ml dry THF where added and the solution was cooled to 0 °C. 155 mg (1.00 mmol, 1 eq) *N*-methoxy-*N*-methyl-3-furancarboxamide dissolved in 5 ml dry THF was added and the mixture stirred at room temperature for 45 min until TLC (Pentan:TBME, 20:1) confirmed the end of the reaction. 20 ml H_2O where added and the phases separated. The aqueous phase was extracted with 3x20 ml ethyl acetate and the combined organic phases where dried over Na_2SO_4. The solvent was removed in vacuo and the crude product purified by flash column chromatography (Pentan:TBME, 20:1). The product was obtained as a colourless oil in 80 % (226 mg, 0.80 mmol) yield. ^{1}H NMR (400 MHz, $CDCl_3$): δ [ppm] = 8.02 – 8.00 (m, 1H), 7.43 (t, J = 1.7 Hz, 1H), 6.76 (dd, J = 1.9, 0.8 Hz, 1H), 3.94 – 3.85 (m, 1H), 2.92 – 2.71 (m, 2H), 1.92 – 1.82 (m, 1H), 1.79 – 1.69 (m, 1H), 1.16 (d, J = 6.1 Hz, 3H), 0.89 (s, 9H), 0.04 (d, J = 8.4 Hz, 6H). ^{13}C-NMR

(100.6 MHz, $CDCl_3$): δ [ppm] = 195.19, 146.98, 144.12, 127.71, 108.66, 67.62, 36.46, 33.60, 25.87, 23.77, 18.08, -4.36, -4.76. (ESI): *m/z* = 305.1547 $[M+Na]^+$ (calculated for $C_{15}H_{26}O_3Si + Na^+$: 305.1543).

4-Ipomeanol

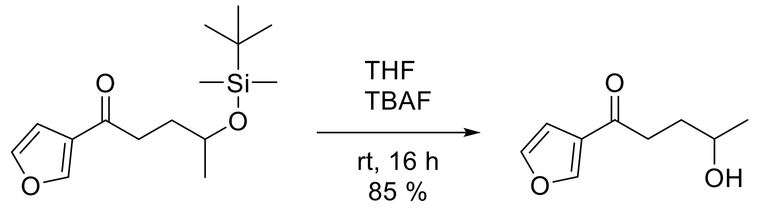

600 mg (2.12 mmol, 1 eq) *O*-TBDMS-4-ipomeanol where dissolved in 5 ml THF. 4.25 ml (4.25 mmol, 2 eq) of a TBAF in THF (1 mol/l) solution where added and the mixture was stirred at room temperature for 16 h. 30 ml saturated NH_4Cl_{aq} where added and the phases separated. The aqueous phase was extracted with 3x30 ml ethyl acetate. The combined organic phases where dried over Na_2SO_4 and the solvent removed in vacuo. The crude product was purified by flash column chromatography (DCM:Acetone; 20:1). The product was obtained as a colorless oil in 85 % (303 mg, 1.80 mmol) yield. ^1H NMR (600 MHz, $CDCl_3$): δ [ppm] = 8.06 (s, 1H), 7.43 (t, J = 1.7 Hz, 1H), 6.78 – 6.75 (m, 1H), 3.95 – 3.82 (m, 1H), 2.91 (t, J = 7.1 Hz, 2H), 1.96 – 1.89 (m, 1H), 1.84 – 1.75 (m, 3H), 1.23 (d, J = 6.2 Hz, 3H). ^{13}C-NMR (100.6 MHz, $CDCl_3$): δ [ppm] = 195.66, 147.45, 144.35, 127.76, 108.75, 77.48, 77.16, 76.84, 67.57, 36.82, 33.10, 23.94. (ESI): *m/z* = 191.0683 $[M+Na]^+$ (calculated for $C_9H_{12}O_3 + Na^+$: 191.0679).
