# Supplementary figures and images for: Functional analysis of CYP4B1 enzymes from apes and humans uncovers evolutionary hot spots for adaptations of the catalytical function
Source: PLoS Genet. 2025 Jun 27;21(6):e1011750. doi: 10.1371/journal.pgen.1011750 (PMC12233900; doi:10.1371/journal.pgen.1011750)

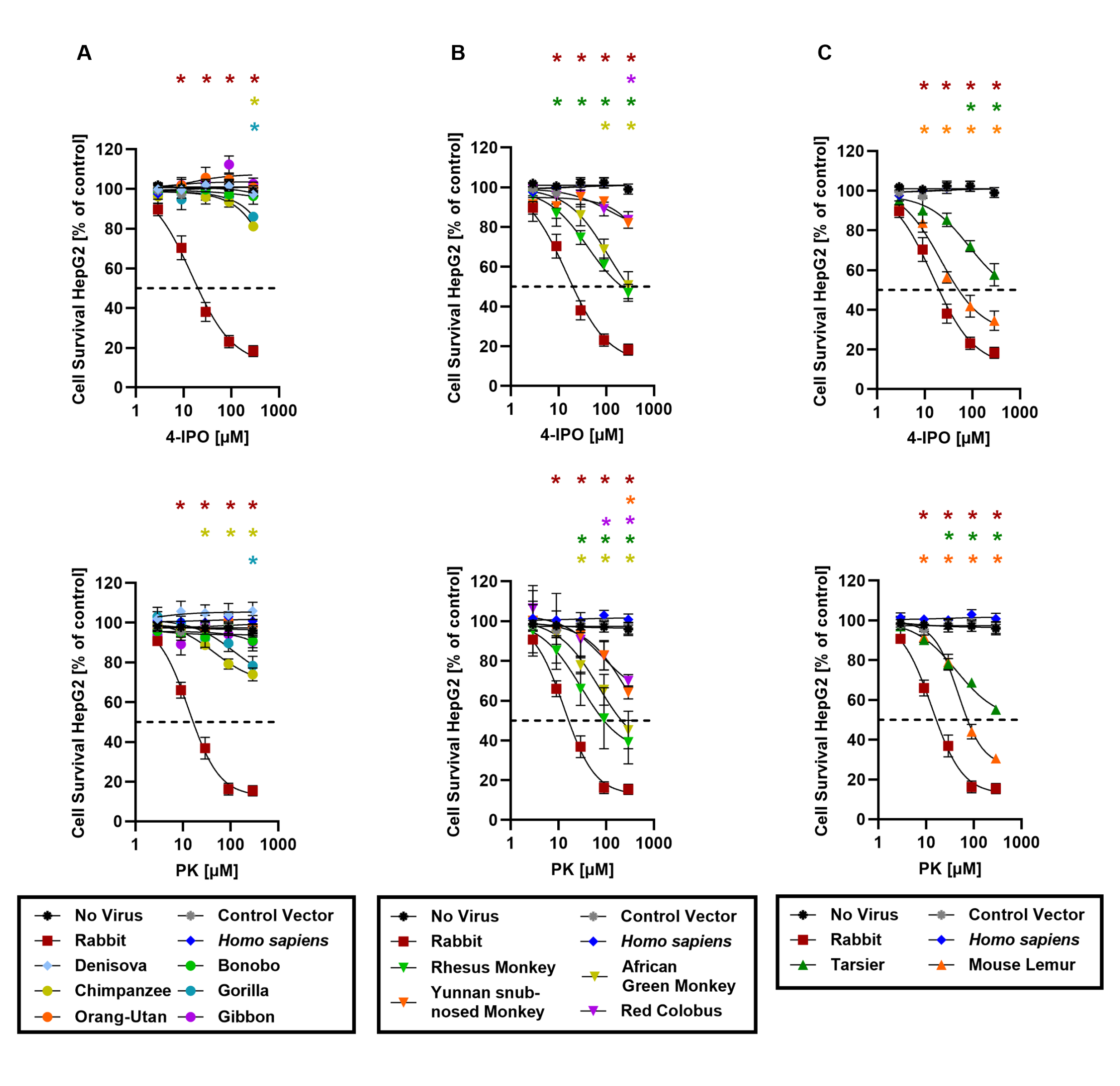

Supplement: S1 Fig — CYP4B1 orthologs from different evolutionary lineages (prosimians, Old World monkeys (OWMs), gibbon, great apes, and Homo sapiens) were systematically analyzed. (A-C) The activity of CYP4B1 isoforms was evaluated performing a MTS assay following a 24 hrs treatment with either 4-ipomeanol, 4-IPO (upper graphs) or Perilla ketone, PK (lower graphs). Experiments were performed in the human liver carcinoma cell lines, HepG2. Graphs are divided based on the evolution of human; left: gibbon and great apes (A), middle: Old World monkeys (B), and right: prosimians (C). For calculation of the half maximal effective concentration reducing cell survival by 50% (GI50 values) a nonlinear fit model was applied to each data set and the bottom was set to 0 while the top was set to 100 (S3 Data). For each data set at least three individual replicates were measured and are shown as mean ± SEM. For statistical analysis, a multiple comparison one-way ANOVA with subsequent Dunnett’s-Post-hoc test was used to determine significant differences between measuring points compared to untreated controls: p-values < 0.05 were defined as significant and were marked with an asterix (*). The underlying data for the graphs in this figure can be found in S2 Data. (TIF) [file pgen.1011750.s003.tif]

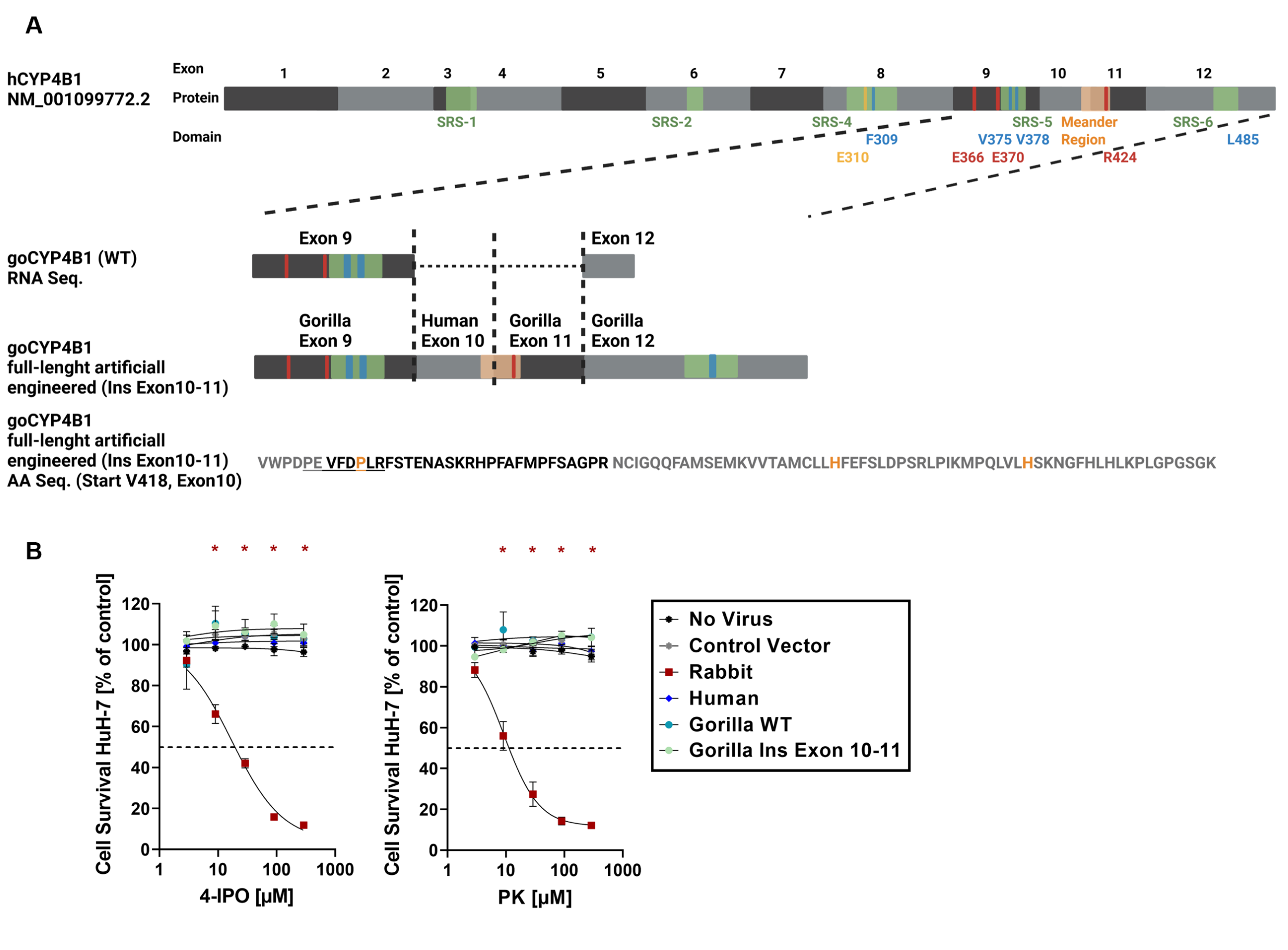

Supplement: S2 Fig — (A) cDNA sequence of wild type gorilla CYP4B1 and engineered gorilla CYP4B1 containing human exon 10 and gorilla exon 10 (corresponding to human exon 11) and full-length gorilla exon 11 (corresponding to human exon 12). The amino acid sequence of the 3’ human exon 10 that is identical in all Hominoidea and gorilla exon 10 (corresponding to human exon 11) that differs in two positions from the human exon (marked in orange). (B) MTS assay of wild type and engineered gorilla CYP4B1 enzymes. For each data set at least three individual replicates were measured and are shown as mean ± SEM. For statistical analysis, a multiple comparison one-way ANOVA with subsequent Dunnett’s-Post-hoc test was used to determine significant differences between measuring points compared to untreated controls: p-values < 0.05 were defined as significant and were marked with an asterix (*). The underlying data for the graphs in this figure can be found in S2 Data. (TIF) [file pgen.1011750.s004.tif]

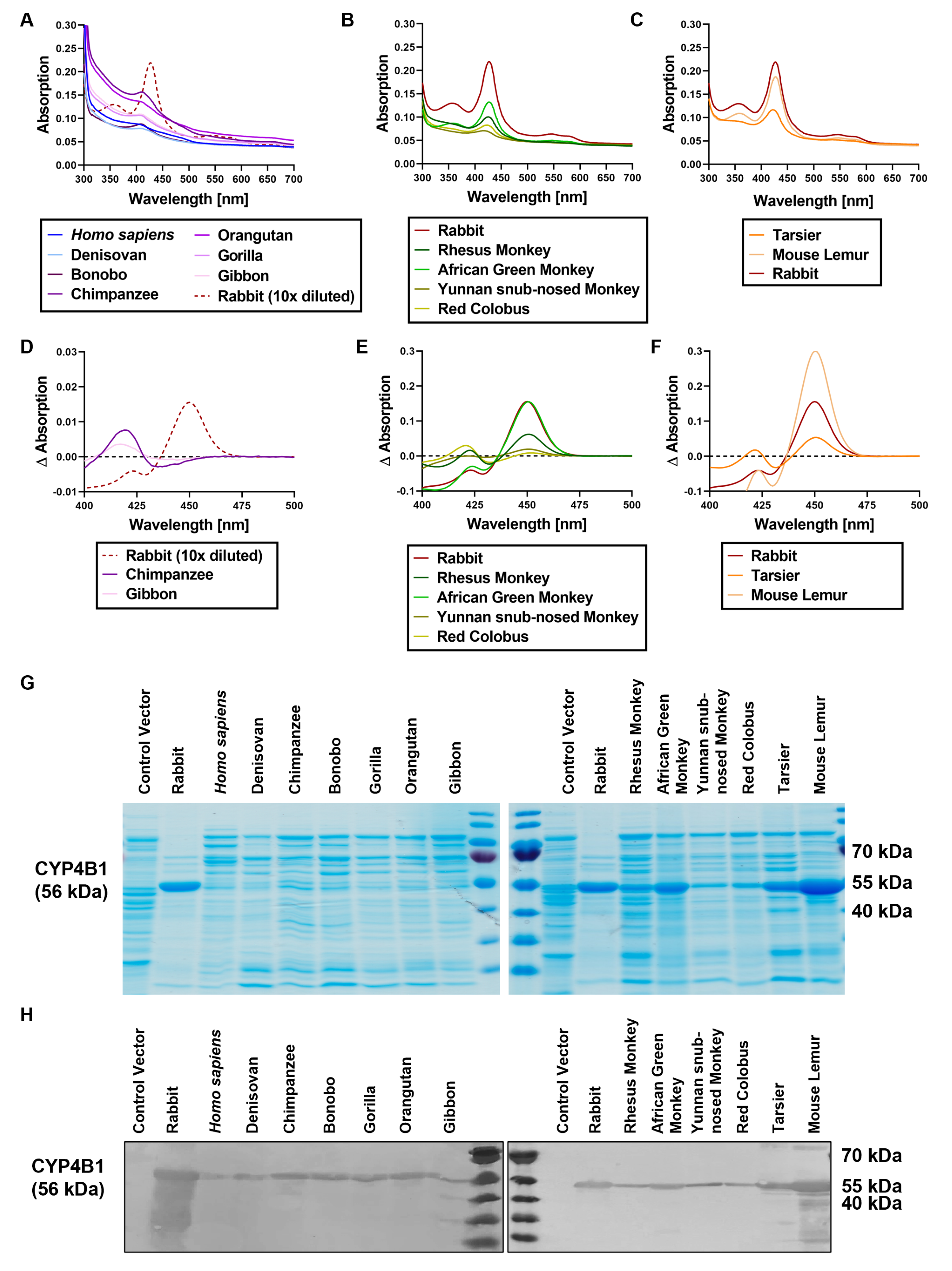

Supplement: S3 Fig — (A-C) Absorption spectra of CYP4B1 orthologs including gibbon, great apes and human (A), OWM (B), and prosimians (C). (D-F). CO-difference spectra of CYP4B1 orthologs purified from soluble protein fractions via affinity chromatography. In contrast to the analyzed OWMs and prosimians CYP4B1 orthologs of Hominoidea revealed no 450 nm peak, thus only the spectra of chimpanzee and gibbon are shown as representatives. (G) SDS-PAGE and (D) Western blot analysis of all CYP4B1 orthologs. Membranes were probed with mouse monoclonal 6x His tag antibody (HIS.H8, 1:1,000 diluted, Thermo Fisher Scientific, #MA1–21315). The secondary horseradish peroxidase-linked goat-anti-mouse polyclonal antibody (1:10,000 diluted, Jackson ImmunoResearch, #115-035-003) was utilized to detect the primary antibody via a colorimetric peroxidase reaction. (TIF) [file pgen.1011750.s005.tif]

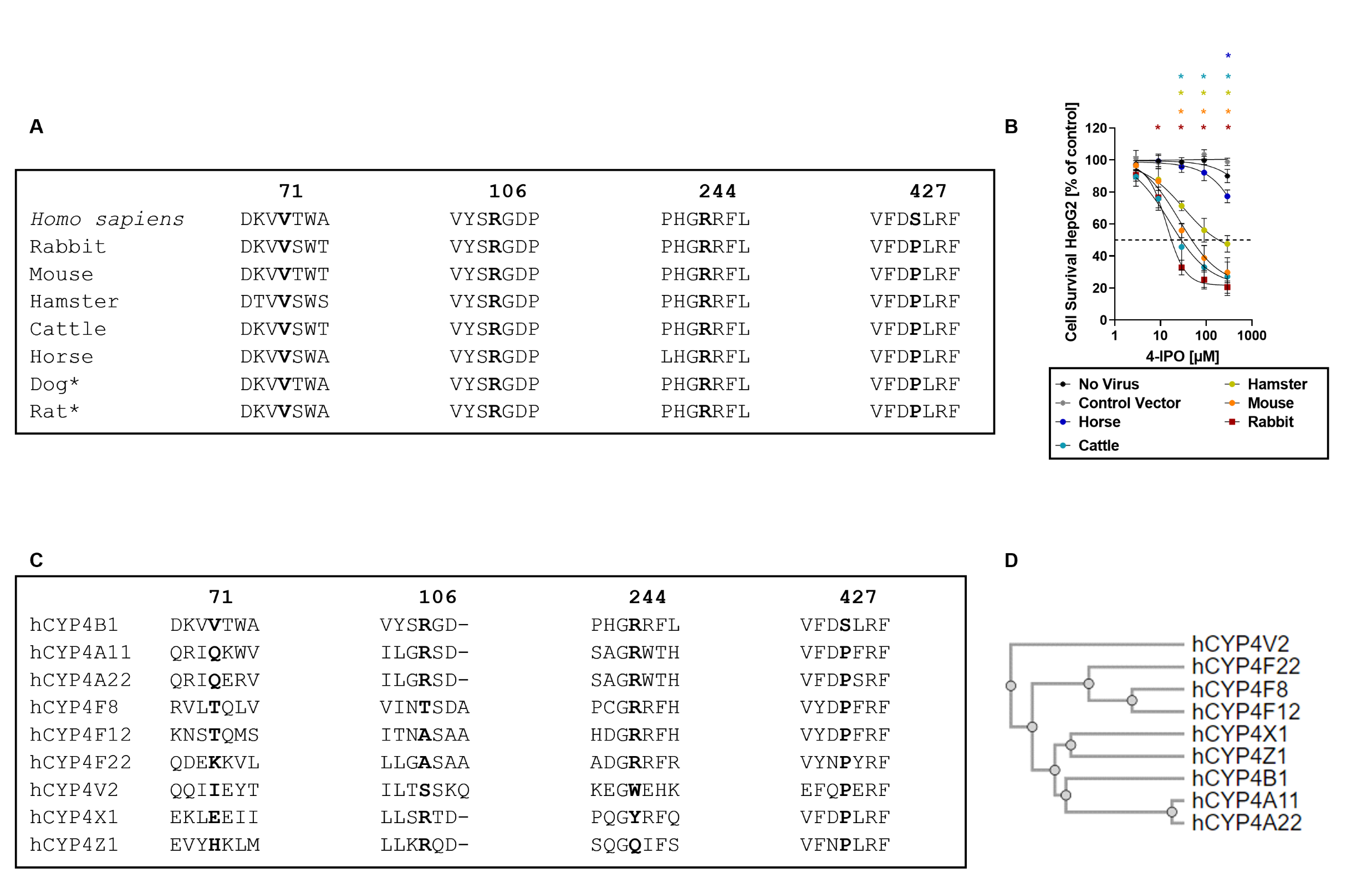

Supplement: S4 Fig — (A) Analyzed were CYP4B1 orthologs from multiple species described to be active. (B) MTS assay of CYP4B1 orthologs described to be active that were tested within our study. (C) On the other hand, members of the CYP4 family were analyzed to identify any conservation patterns of the identified key positions relevant for enzyme function. (D) Guide tree of human CYP4 enzymes created with ClustalW. The underlying data for the graphs in this figure can be found in S2 Data and the respective GI50 values for each CYP4B1 ortholog can be found in S3 Data. (TIF) [file pgen.1011750.s006.tif]

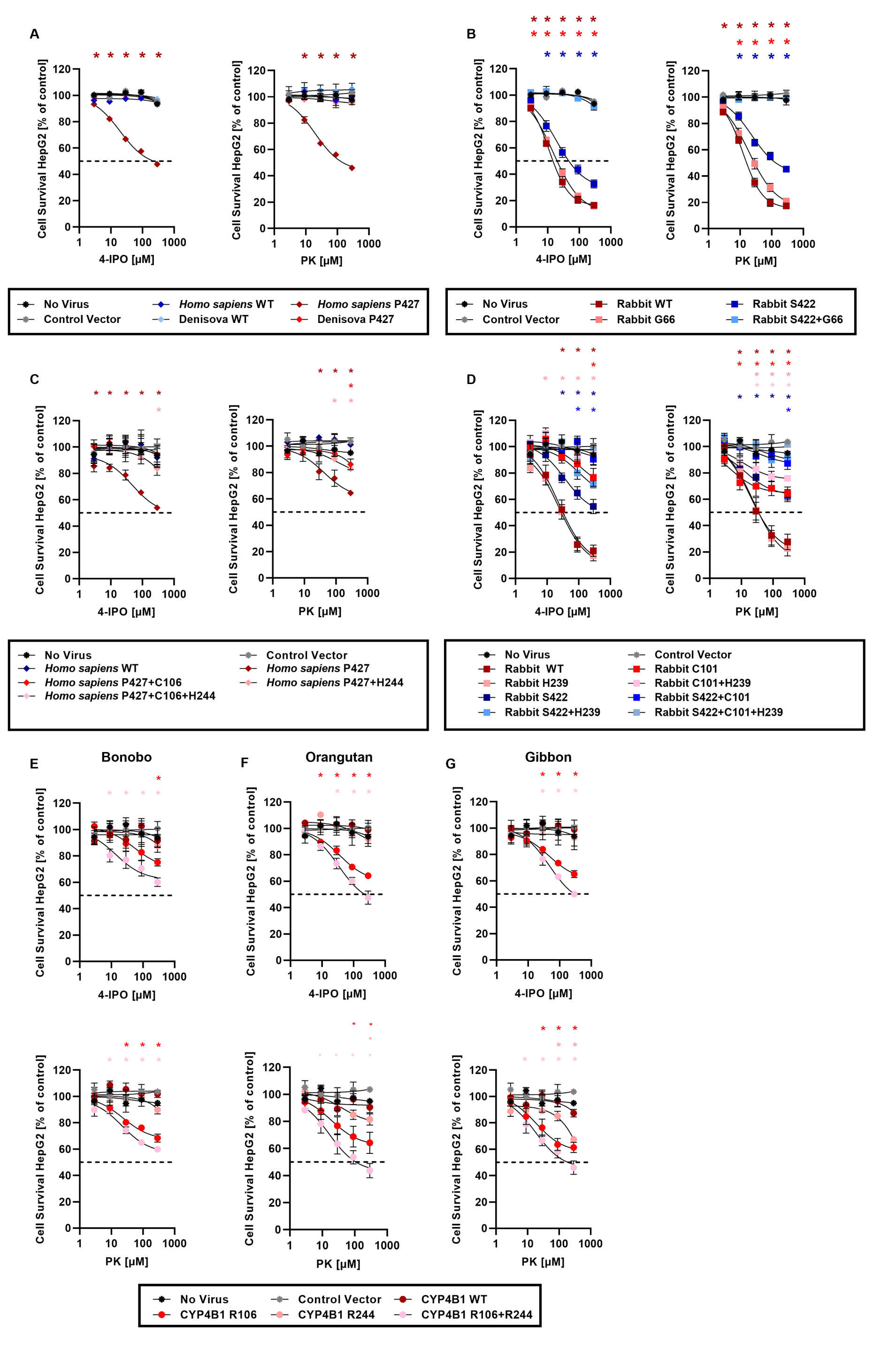

Supplement: S5 Fig — (A) Activities of Homo sapiens wild type (S427; WT) and ‘re-activated’ variant p.S427P (P427), as well as Denisovan wild type (S427) and Denisovan mutant p.S427P. (B) p.V66G was introduced and analyzed in rabbit wild type (P422; WT) and mutant p.P422S (S422). (C) In addition, p.R106C and p.R244H were systematically analyzed in Homo sapiens, (D) rabbit (corresponding to p.R101C and pR239H), and exemplified great apes (bonobo (E) and orangutan (F), and gibbon (G)). Bioactivation of 4-IPO or PK was measured via MTS assay following a 24 hrs treatment; based on a nonlinear fit the respective GI50 values were calculated (S3 Data). All experiments were performed with a minimum of three individual replicates. For each data set at least three individual replicates were measured and are shown as mean ± SEM. For statistical analysis, a multiple comparison one-way ANOVA with subsequent Dunnett’s-Post-hoc test was used to determine significant differences between measuring points compared to untreated controls: p-values < 0.05 were defined as significant and were marked with an asterix (*). The underlying data for the graphs in this figure can be found in S2 Data. (TIF) [file pgen.1011750.s007.tif]

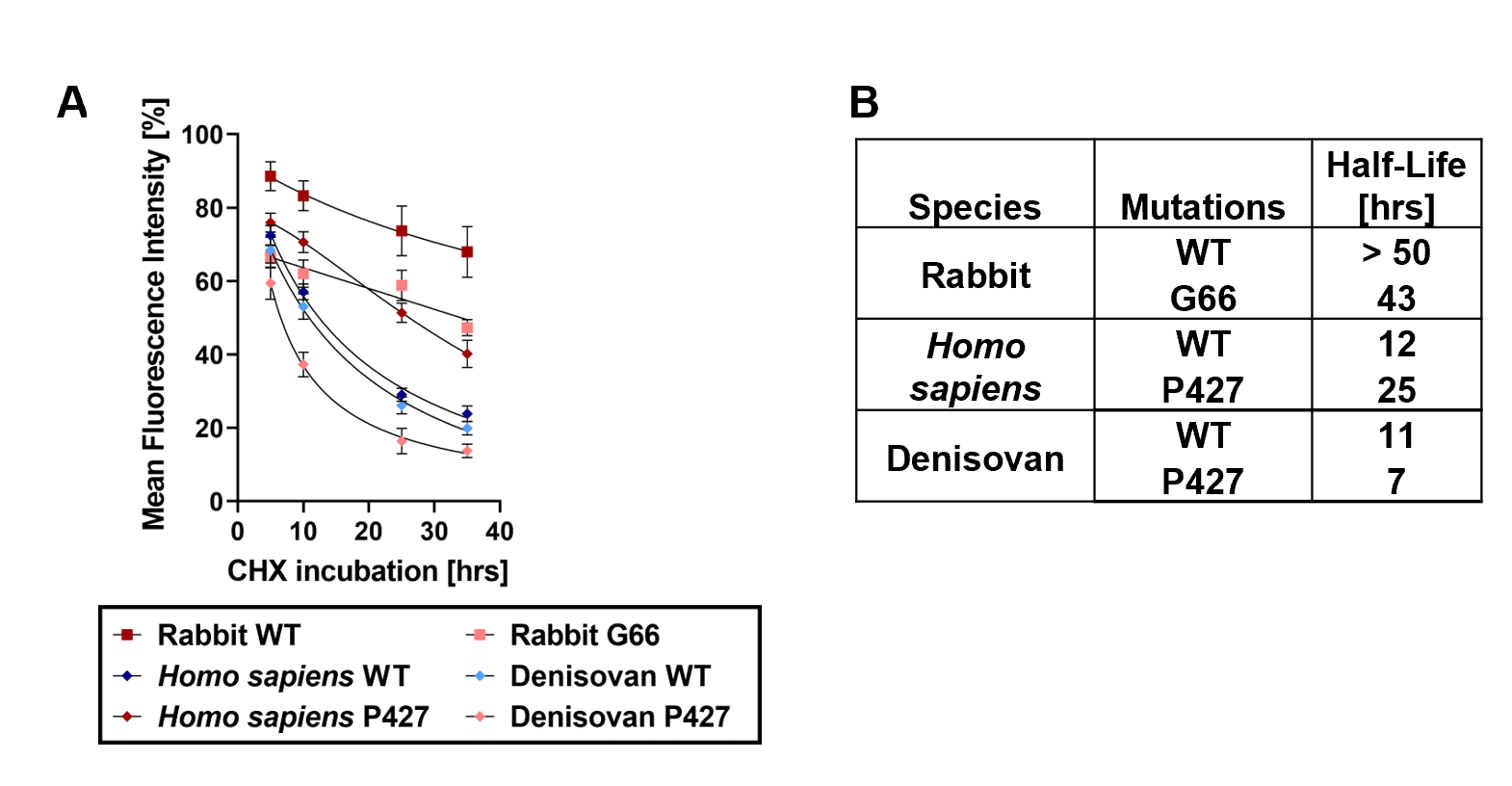

Supplement: S6 Fig — (A) FACS measurements to analyze any effects of the p.V71G (corresponding to p.V66G in rabbit) on protein stability. (B) Calculated half-life of the respective proteins. The underlying data for the graphs in this figure can be found in S2 Data. (TIF) [file pgen.1011750.s008.tif]

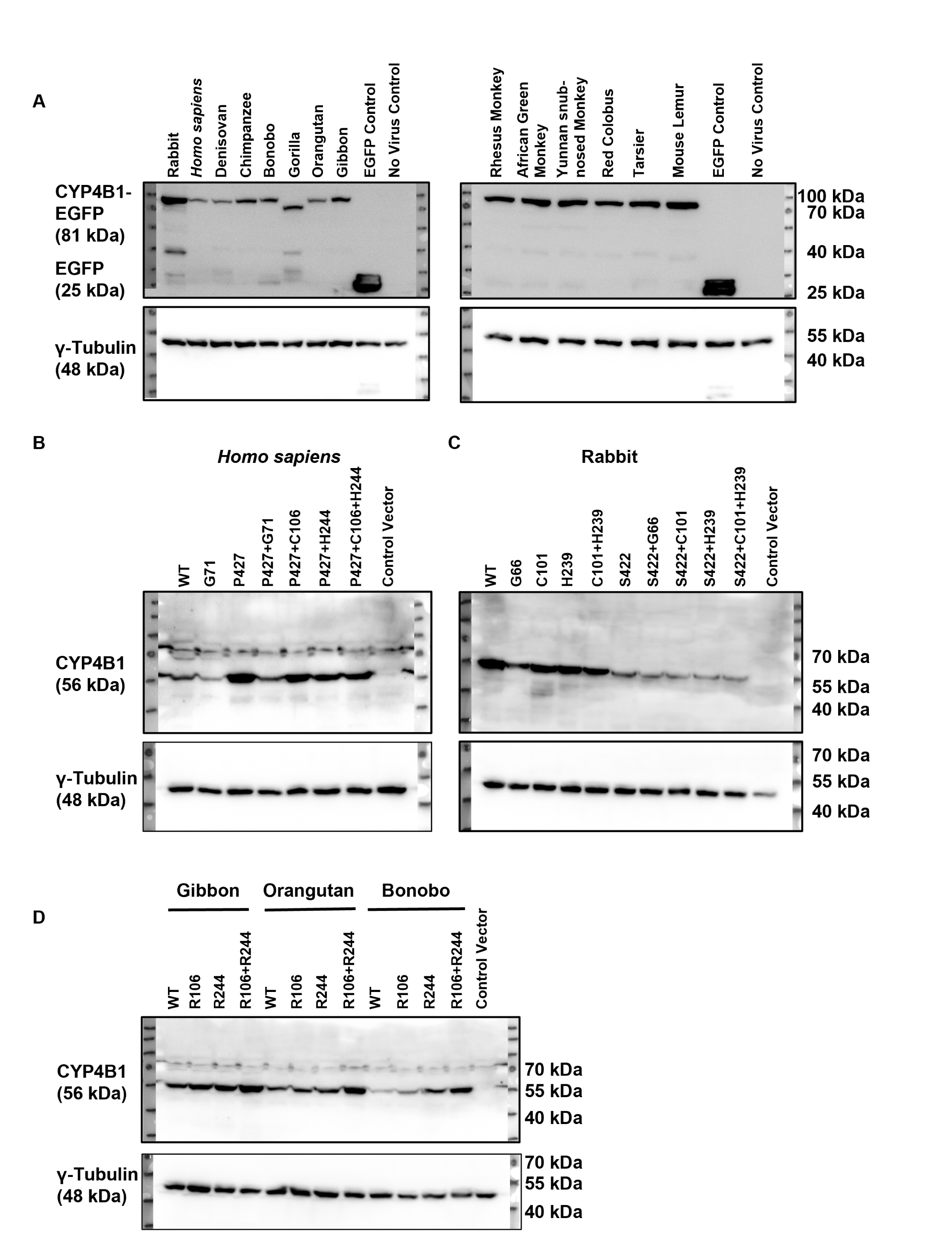

Supplement: S7 Fig — (A) Western Blots of CYP4B1-EGFP fusion proteins. For detection, an anti-EGFP antibody was used. (B) Effect of the amino acid exchanges p.P427S, p.V71G, p.R106C and p.R244H on the expression of Homo sapiens and (C) rabbit (wild type and p.P422S variant) CYP4B1. Human CYP4B1 enzymes were detected with an anti-human CYP4B1 polyclonal antibody. Rabbit CYP4B1 enzymes were detected with an anti-rabbit CYP4B1 polyclonal antibody. (D) Effect of the amino acid exchanges p.C106R and p.H244R on expression levels of gibbon, orangutan, and bonobo CYP4B1 proteins in HuH-7 cells. The hominoid proteins were detected with an anti-human CYP4B1 antibody. γ-tubulin served as a loading control for all performed western blots. (TIF) [file pgen.1011750.s009.tif]
