# Supplementary material for: Functional analysis of CYP4B1 enzymes from apes and humans uncovers evolutionary hot spots for adaptations of the catalytical function
Source: PLoS Genet. 2025 Jun 27;21(6):e1011750. doi: 10.1371/journal.pgen.1011750 (PMC12233900; doi:10.1371/journal.pgen.1011750)
Supplement: S1 Text — (PDF) [file pgen.1011750.s015.pdf]

Detailed description of the sequence analysis of gorilla CYP4B1

However, the amino acid sequence alignment revealed a premature stop at position 410 in the gorilla *CYP4B1* ortholog (**S1 File**). We therefore extracted genomic DNA from the lung tissues of the three gorilla individuals and performed PCR with forward primers located in exon 9 and reverse primers in exons 10 and 11, respectively. Sanger-sequencing of the PCR products showed that the exon 10 sequence present in human (NM_00131916.1) and great apes (e.g., the chimpanzee) was missing in all six alleles of the three gorillas analyzed, due to a deletion in the two introns that originally may have surrounded this exon in the gorilla genome (**Fig 2A**). This loss of exon 10 and the presence of exons 11 and 12 in the genomic sequence of the gorilla seems to be correctly mirrored in the Ensembl transcript (ENSGGOT00000016915.3) containing 490 amino acids. However, a closer look at this transcript revealed a misannotation in the splice donor of the gorilla exon 9 by -1 nucleotide, therefore not correctly showing the amino acid sequence that is translated in cells. In addition, our mRNA sequencing of the primary lung tissues of the animals demonstrated that exon 10 (corresponding to exon 11 in human and chimpanzee) is also not included in the main transcript of the gorilla CYP4B1 (**Fig 2B**). Therefore, we analyzed the strength of the splice donor and acceptor sites of exons 9 to 11/12 of the gorilla, *Homo sapiens*, and chimpanzee *CYP4B1* genes using the MAXENT (https://pubmed.ncbi.nlm.nih.gov/15285897/) or HBS (https://pubmed.ncbi.nlm.nih.gov/14627829/) algorithms. The analyses revealed a strong splice acceptor in the 5' part of exon 10 (MAXENT score of 11.3 or 10.9 for human and chimpanzee CYP4B1, respectively) and a much weaker splice acceptor (MAXENT score of 5.9) for the gorilla exon 10 and the corresponding human and chimpanzee exon 11. Therefore, exon 10 (corresponding to human exon 11) is largely skipped in the gorilla mRNA, leading to a frameshift in the last exon that codes for a protein of 410 amino acids.
